# Supplementary material for: Intrinsic rewards explain context-sensitive valuation in reinforcement learning
Source: PLoS Biol. 2023 Jul 17;21(7):e3002201. doi: 10.1371/journal.pbio.3002201 (PMC10374061; doi:10.1371/journal.pbio.3002201)
Supplement: S1 Table — Previously collected data sets were originally reported by [12] (B21), [11] (B18), [14] (G12), [13] (B22). For each data set experiment we used (“Exp.”), we report: the rewards and probabilities associated with each context and bandit, as well as their expected value (EV); the key comparisons for which participants show irrational behavior, or for which models make specific predictions; whether feedback was partial or complete (i.e., including counterfactual) during learning; whether feedback was partial, complete, or absent during testing; whether bandit pairs were presented in a blocked or interleaved manner; whether there was a difference in the absolute magnitude of reward across bandit pairs (“Mag. Δ”); whether the task included negative outcomes (“Loss”); and the total number of participants in each original experiment (“N”). (PDF) [file pbio.3002201.s001.pdf]

Name	Exp.	Learn contingencies (EV)	Key comp.	Learn feedback	Test feedback	Order	Mag. Δ	Loss	N
**B21**	1	• Context 1<br>– 1: 75% 10, 25% 0 (7.5)<br>– 2: 25% 10, 75% 0 (2.5)<br>• Context 2<br>– 3: 75% 10, 25% 0 (7.5)<br>– 4: 25% 10, 75% 0 (2.5)<br>• Context 3<br>– 5: 75% 1, 25% 0 (0.75)<br>– 6: 25% 1, 75% 0 (0.25)<br>• Context 4<br>– 7: 75% 1, 25% 0 (0.75)<br>– 8: 25% 1, 75% 0 (0.25)	Bandit 4 vs. 7	Partial	None	Interleaved	Yes	No	100
	2	”	”	Partial	Partial	Interleaved	”	”	”
	3	”	”	Complete	None	Interleaved	”	”	”
	4	”	”	Complete	Complete	Interleaved	”	”	”
	5	”	”	Partial	None	Blocked	”	”	”
	6	”	”	Partial	Partial	Blocked	”	”	”
	7	”	”	Complete	None	Blocked	”	”	”
	8	”	”	Complete	Complete	Blocked	”	”	”
**B18**	1	• Context 1<br>– 1: 75% 1, 25% 0 (0.75)<br>– 2: 25% 1, 75% 0 (0.25)<br>• Context 2<br>– 3: 75% 0.1, 25% 0 (0.075)<br>– 4: 25% 0.1, 75% 0 (0.025)<br>• Context 3<br>– 5: 25% -1, 75% 0 (-0.25)<br>– 6: 75% -1, 25% 0 (-0.75)<br>• Context 4<br>– 7: 25% -0.1, 75% 0 (-0.025)<br>– 8: 75% -0.1, 25% 0 (-0.075)	Bandit 2 vs. 3<br>Bandit 2 vs. 5<br>Bandit 4 vs. 5<br>Bandit 4 vs. 7	Partial	None	Interleaved	Yes	Yes	20
	2	”	”	Complete	”	”	”	”	40
**G12**	1	• Context 1<br>– 1: 90% 1, 10% 0 (0.9)<br>– 2: 10% 1, 90% 0 (0.1)<br>• Context 2<br>– 3: 80% 1, 20% 0 (0.8)<br>– 4: 20% 1, 80% 0 (0.2)<br>• Context 3<br>– 5: 10% -1, 90% 0 (-0.1)<br>– 6: 90% -1, 10% 0 (-0.9)<br>• Context 4<br>– 7: 20% -1, 80% 0 (-0.2)<br>– 8: 80% -1, 20% 0 (-0.8)	Bandit 2 vs. 5<br>Bandit 2 vs. 7<br>Bandit 4 vs. 5<br>Bandit 4 vs. 7	Partial	None	Interleaved	No	Yes	75

Name	Exp.	Learn contingencies (EV)	Key comp.	Learn feedback	Test feedback	Order	Mag. Δ	Loss	N
**B22**	1	• Context 1<br>– 1: 100% 14 ± 2 (14)<br>– 2: 100% 50 ± 2 (50)<br>• Context 2<br>– 3: 100% 14 ± 2 (14)<br>– 4: 100% 32 ± 2 (32)<br>– 5: 100% 50 ± 2 (50)<br>• Context 3<br>– 6: 100% 14 ± 2 (14)<br>– 7: 100% 86 ± 2 (86)<br>• Context 4<br>– 8: 100% 14 ± 2 (14)<br>– 9: 100% 50 ± 2 (50)<br>– 10: 100% 86 ± 2 (86)	Bandit 2 vs. 5<br>Bandit 2 vs. 10<br>Bandit 7 vs. 5<br>Bandit 7 vs. 10	Complete	None	Interleaved	Yes	No	50
**M22**	1	• Context 1<br>– 1: 100% 14 ± 2 (14)<br>– 2: 100% 50 ± 2 (50)<br>– 3: 100% 86 ± 2 (86)<br>• Context 2<br>– 4: 100% 14 ± 2 (14)<br>– 5: 100% 50 ± 2 (50)<br>– 6: 100% 86 ± 2 (86)	Bandit 2 vs. 5<br>Bandit 2 vs. 1/4<br>Bandit 5 vs. 1/4<br>Bandit 2 vs. 3/6<br>Bandit 5 vs. 3/6	Complete	None	Interleaved<br>Presentation times:<br>• Context 1<br>– Bandit 1 vs. 2 vs. 3: 20<br>– Bandit 1 vs. 2: 0<br>– Bandit 1 vs. 3: 20<br>– Bandit 2 vs. 3: 20<br>• Context 2<br>– Bandit 4 vs. 5 vs. 6: 20<br>– Bandit 4 vs. 5: 0<br>– Bandit 4 vs. 6: 20<br>– Bandit 5 vs. 6: 20	Yes	No	50
