# Supplementary figures and images for: Intrinsic rewards explain context-sensitive valuation in reinforcement learning
Source: PLoS Biol. 2023 Jul 17;21(7):e3002201. doi: 10.1371/journal.pbio.3002201 (PMC10374061; doi:10.1371/journal.pbio.3002201)

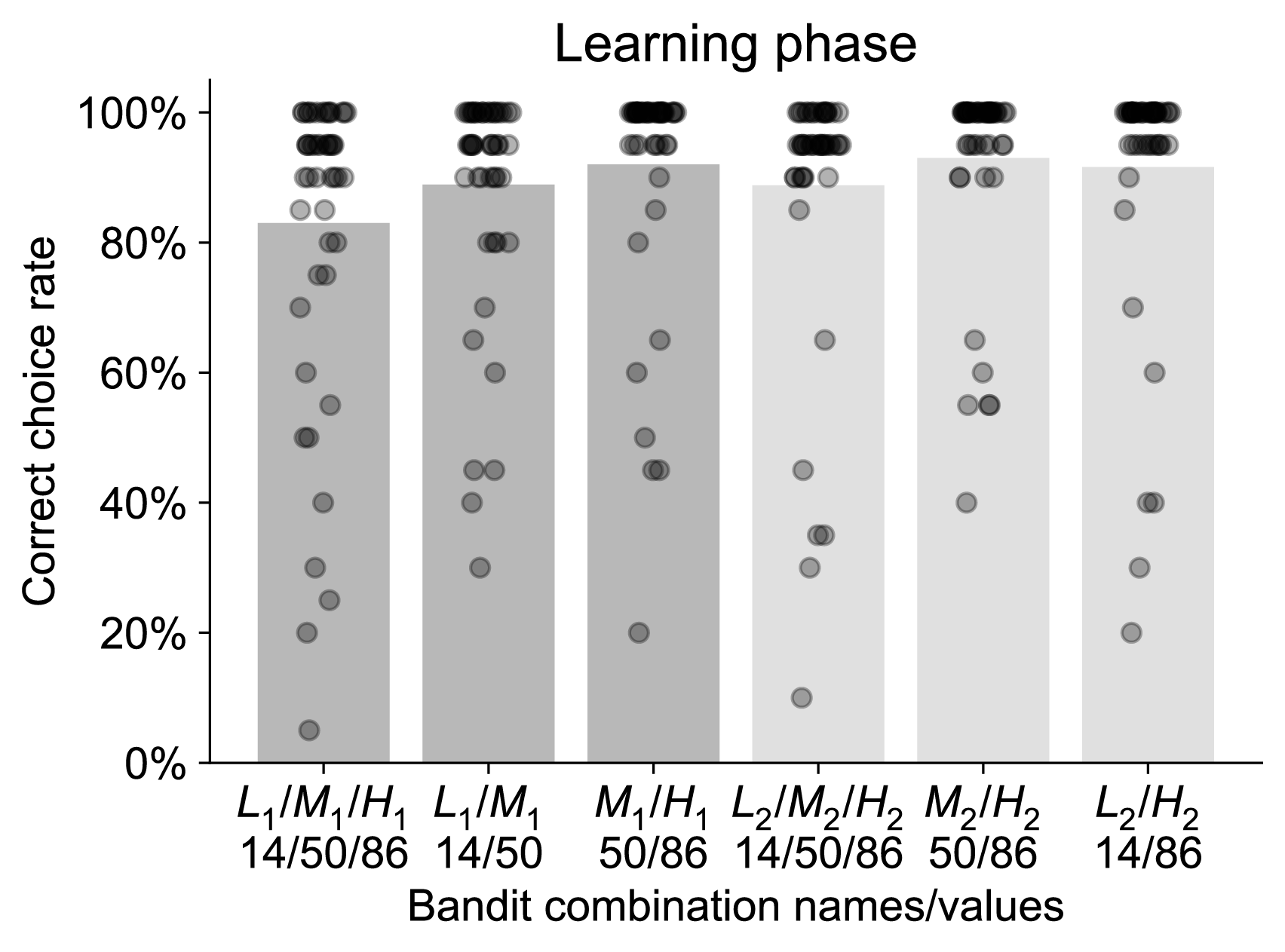

Supplement: S1 Fig — Participants learned to discriminate the correct option across bandit combinations in the learning phase. Data and analysis scripts underlying this figure are available at https://osf.io/sfnc9/. (TIF) [file pbio.3002201.s002.tif]

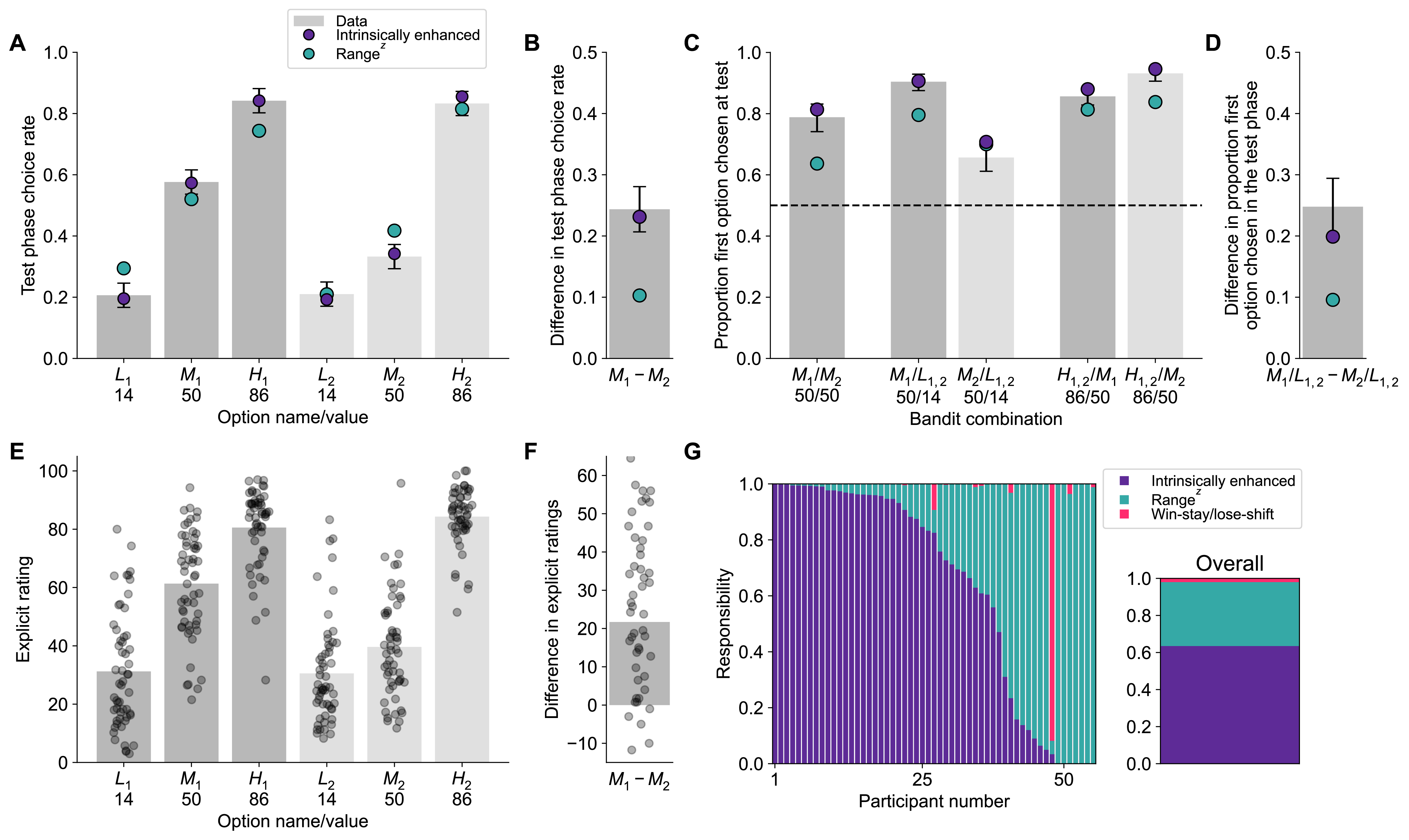

Supplement: S2 Fig — As in the main study (M22), behavioral results and computational modeling support the intrinsically enhanced model. (A) During the test phase, the mid-value option of context 1 (darker gray) was chosen more often than the mid-value option of context 2 (lighter gray), a pattern that was also evident in the intrinsically enhanced model’s, but not the rangez model’s behavior. (B) Difference in test phase choice rates between stimulus M1 and M2. (C) When the 2 mid-value options were pitted against each other, participants preferred the one from context 1. When either was pitted against a low-value option, participants selected the mid-value option from context 1 more often than the mid-value option from context 2. When either was pitted against a high-value option, participants selected the high-value option from context 1 less often than the high-value option from context 2. The dotted line indicates chance level (0.5). All these behavioral signatures were captured by the intrinsically enhanced, but not the range adaptation model. (D) Difference between M1 and M2 in the proportion of times the option was chosen when compared to either L1 or L2. (E) Participants explicitly reported the mid-value option of context 1 as having a higher value than the mid-value option of context 2. (F) Differences in explicit ratings between option M1 and M2. (G) Model fitting favored the intrinsically enhanced model. Data and analysis scripts underlying this figure are available at https://osf.io/sfnc9/. (TIF) [file pbio.3002201.s003.tif]

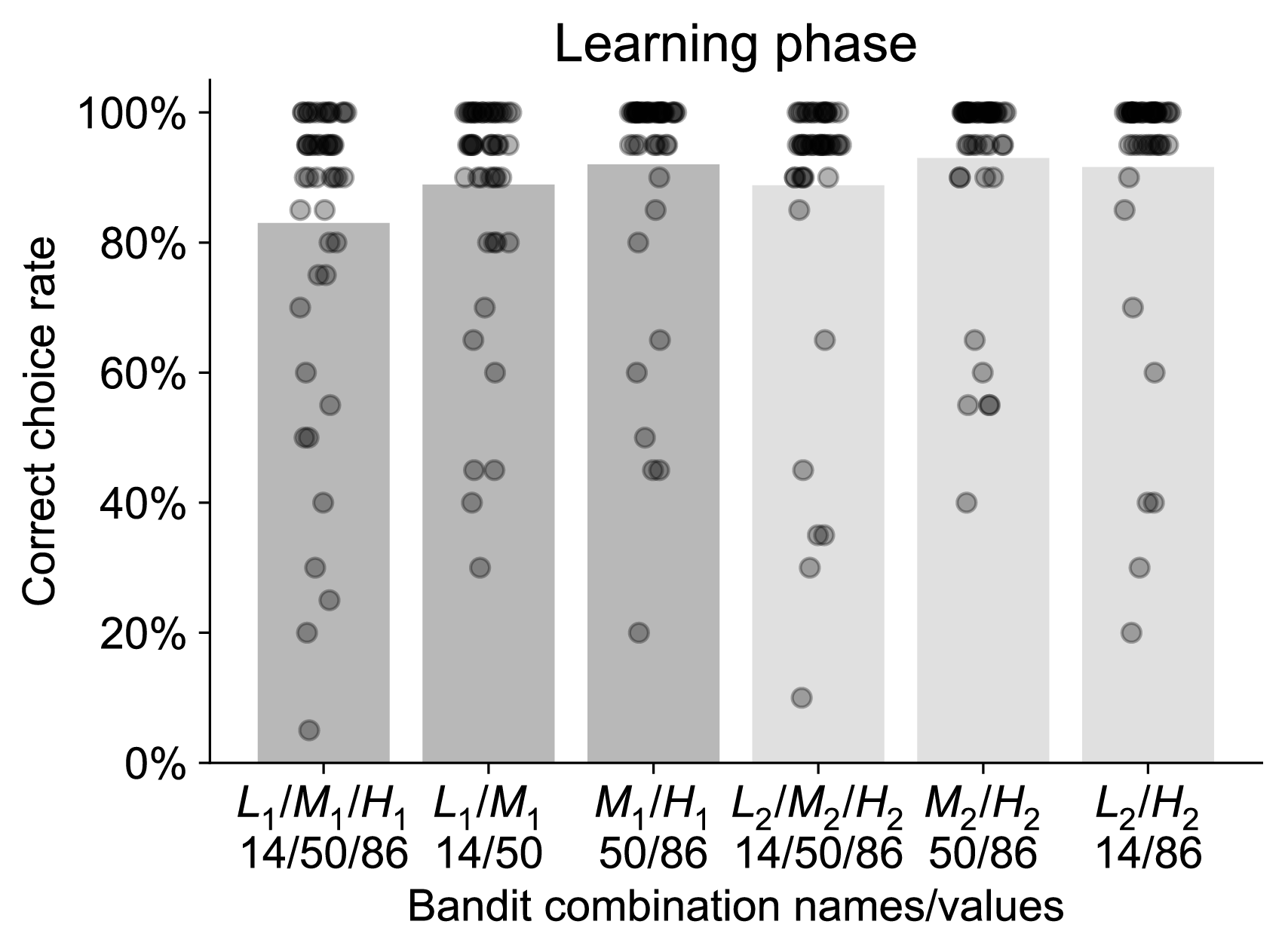

Supplement: S3 Fig — Participants learned to discriminate the correct option across bandit combinations in the learning phase. Data and analysis scripts underlying this figure are available at https://osf.io/sfnc9/. (TIF) [file pbio.3002201.s004.tif]

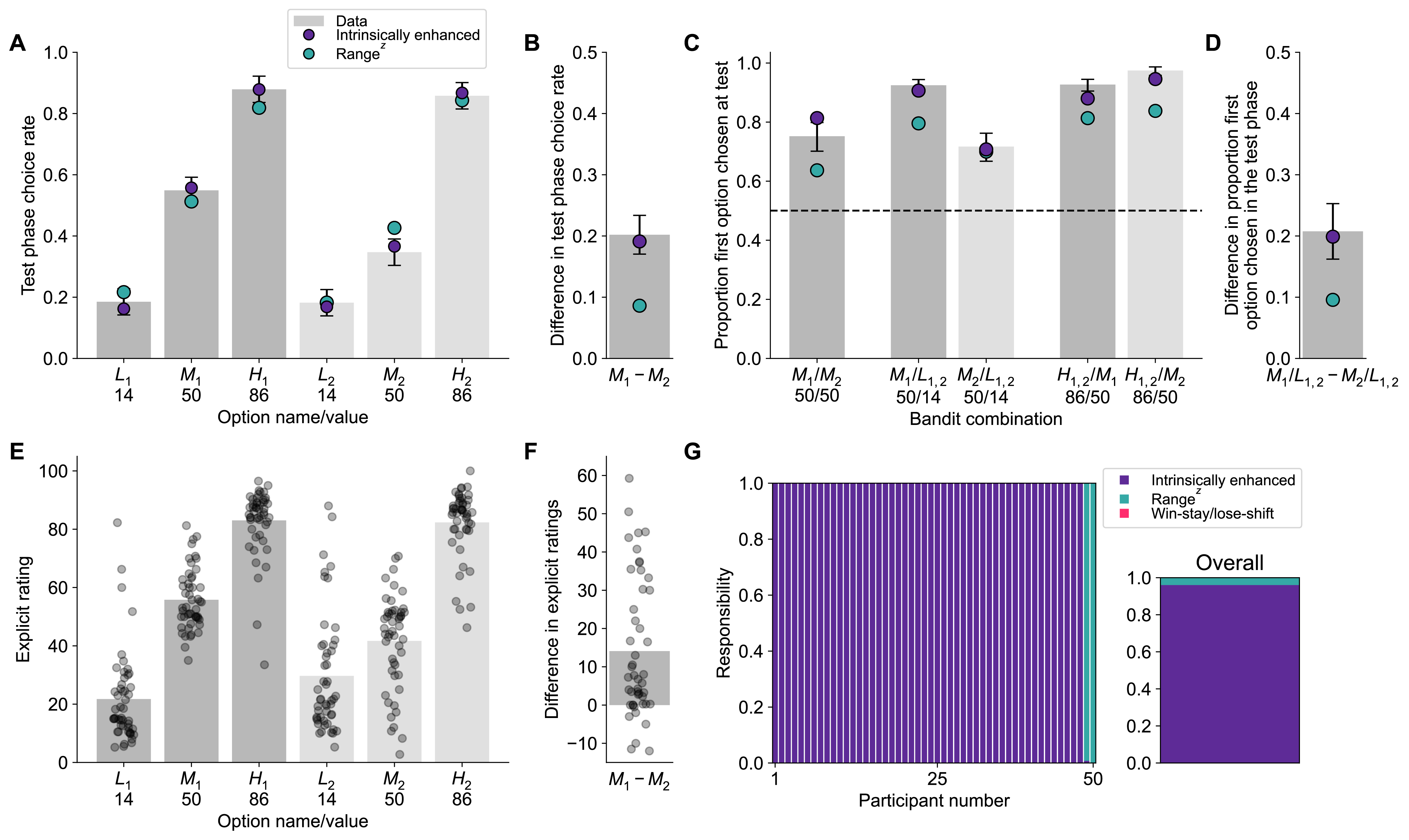

Supplement: S5 Fig — As in the main study (M22) and the replication (M22R), behavioral results and computational modeling support the intrinsically enhanced model. See S2 Fig for caption details. Data and analysis scripts underlying this figure are available at https://osf.io/sfnc9/. (TIF) [file pbio.3002201.s006.tif]

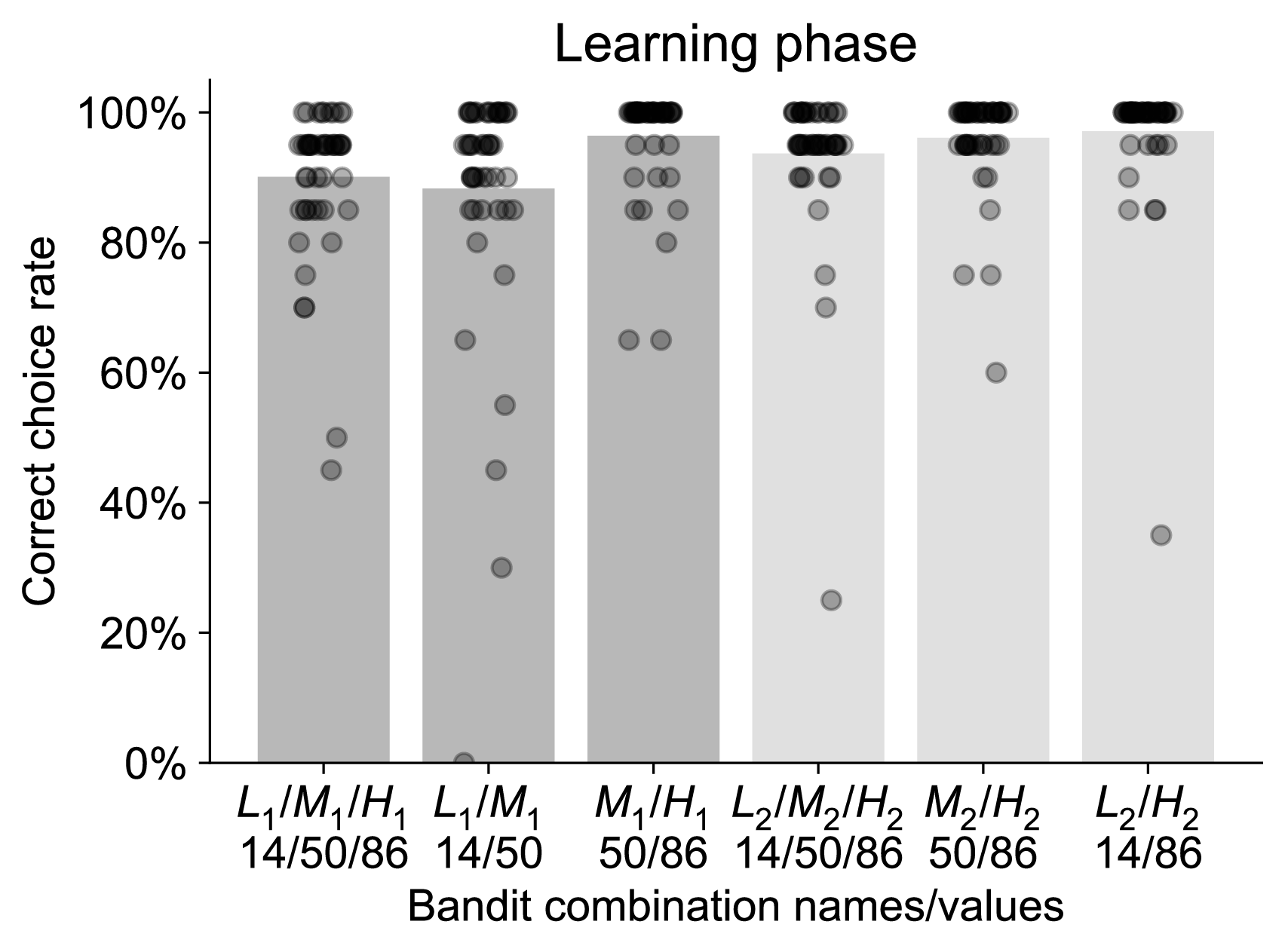

Supplement: S6 Fig — Participants learned to discriminate the correct option across bandit combinations in the learning phase. Data and analysis scripts underlying this figure are available at https://osf.io/sfnc9/. (TIF) [file pbio.3002201.s007.tif]

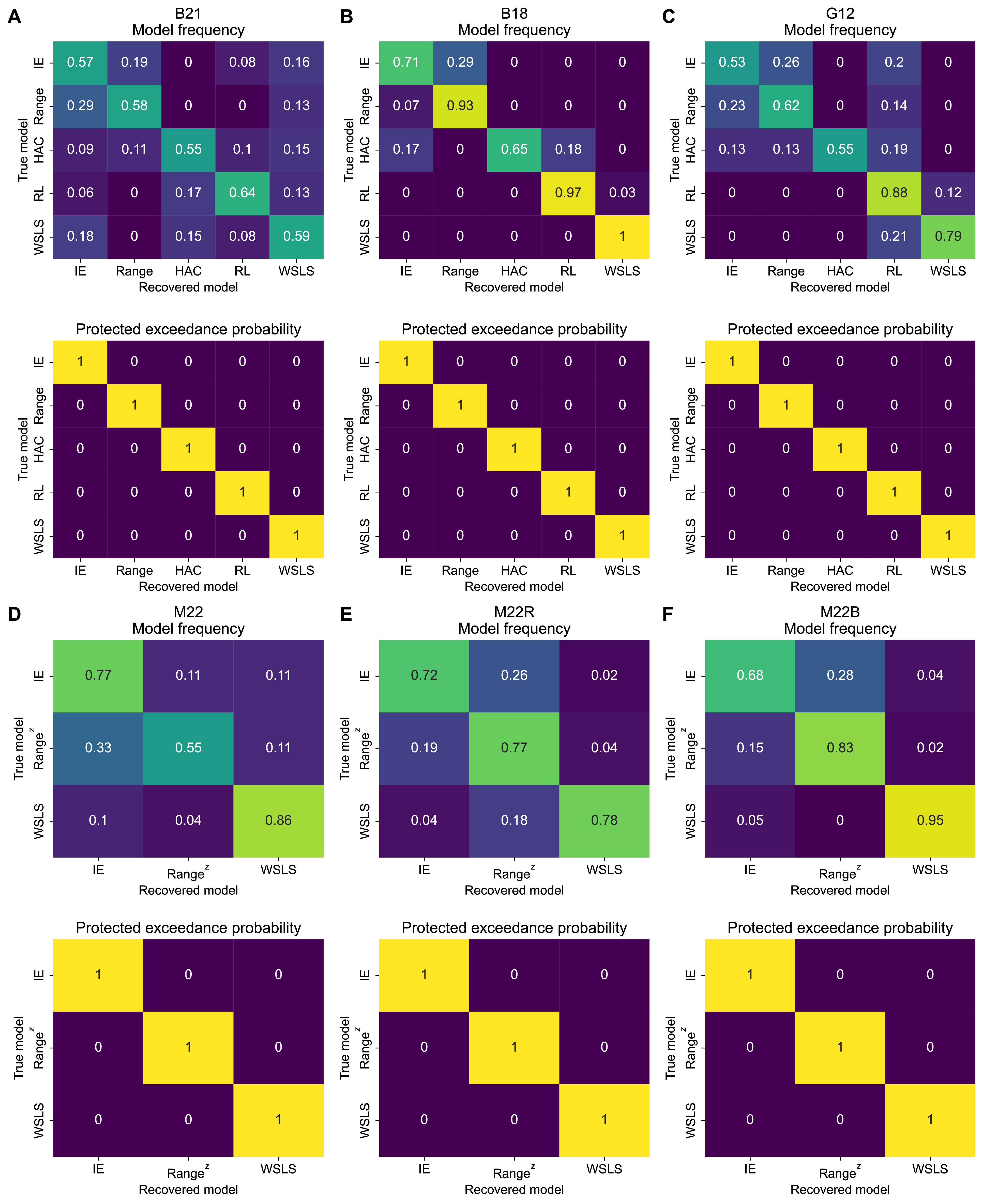

Supplement: S7 Fig — The upper row in each subplot shows model frequencies, the lower row shows protected exceedance probabilities. Model name abbreviations: IE = intrinsically enhanced, HAC = hybrid actor-critic, RL = unbiased, WSLS = win-stay/lose-shift. Data and analysis scripts underlying this figure are available at https://osf.io/sfnc9/. (TIF) [file pbio.3002201.s008.tif]

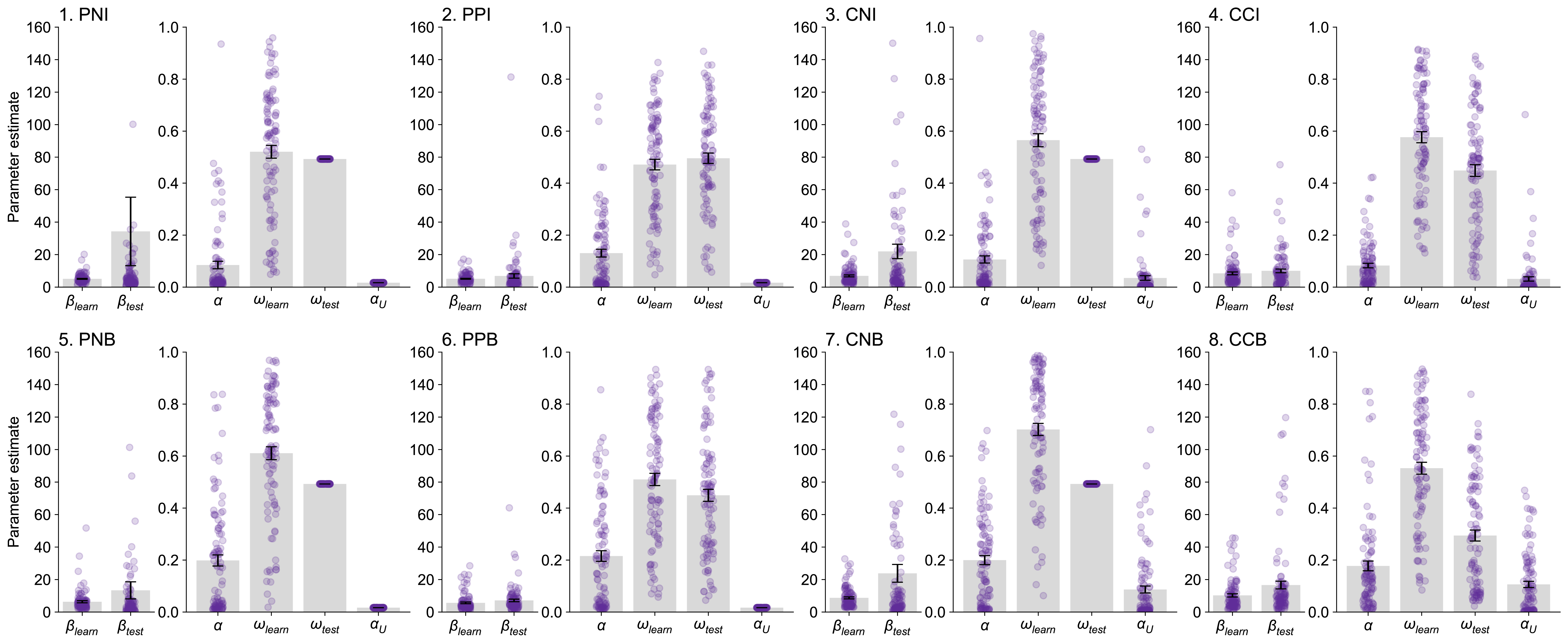

Supplement: S8 Fig — Note that αu could only be fit in experiments with counterfactual feedback, and ωtest could only be fit in experiments with counterfactual feedback at testing, and are therefore left at the initial prior. Abbreviations: the first letter in each triplet indicates whether feedback was partial (P) or complete (C) during learning; the second letter indicates whether feedback in the test phase was partial (P), complete (C), or not provided (N); the third letter indicates whether the experimental design was interleaved (I) or blocked (B). Error bars indicate the SEM. (B) Model responsibilities overall and across experimental conditions. Computational modeling scripts used to produce this figure are available at https://osf.io/sfnc9/. (TIF) [file pbio.3002201.s009.tif]

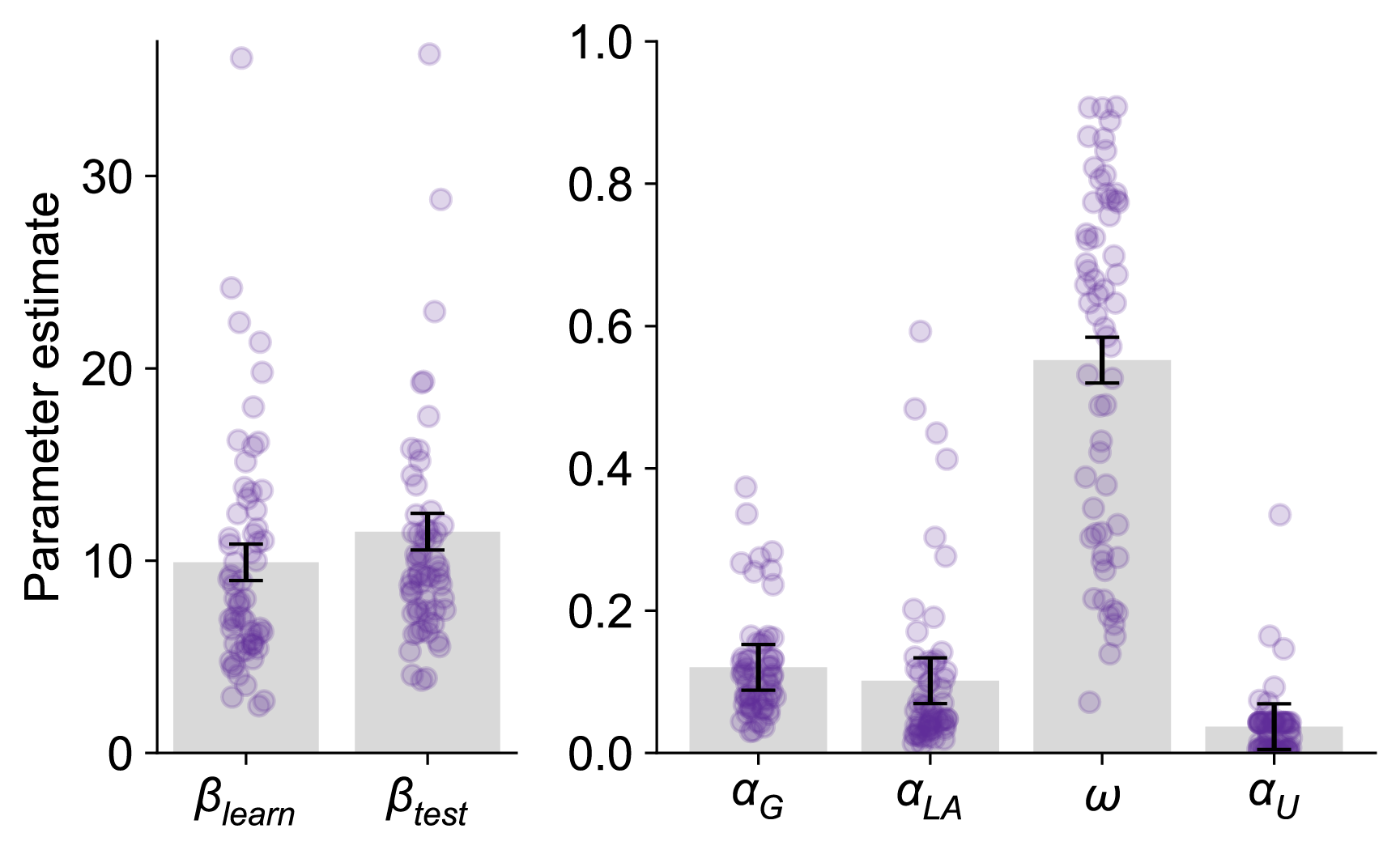

Supplement: S9 Fig — Computational modeling scripts used to produce this figure are available at https://osf.io/sfnc9/. (TIF) [file pbio.3002201.s010.tif]

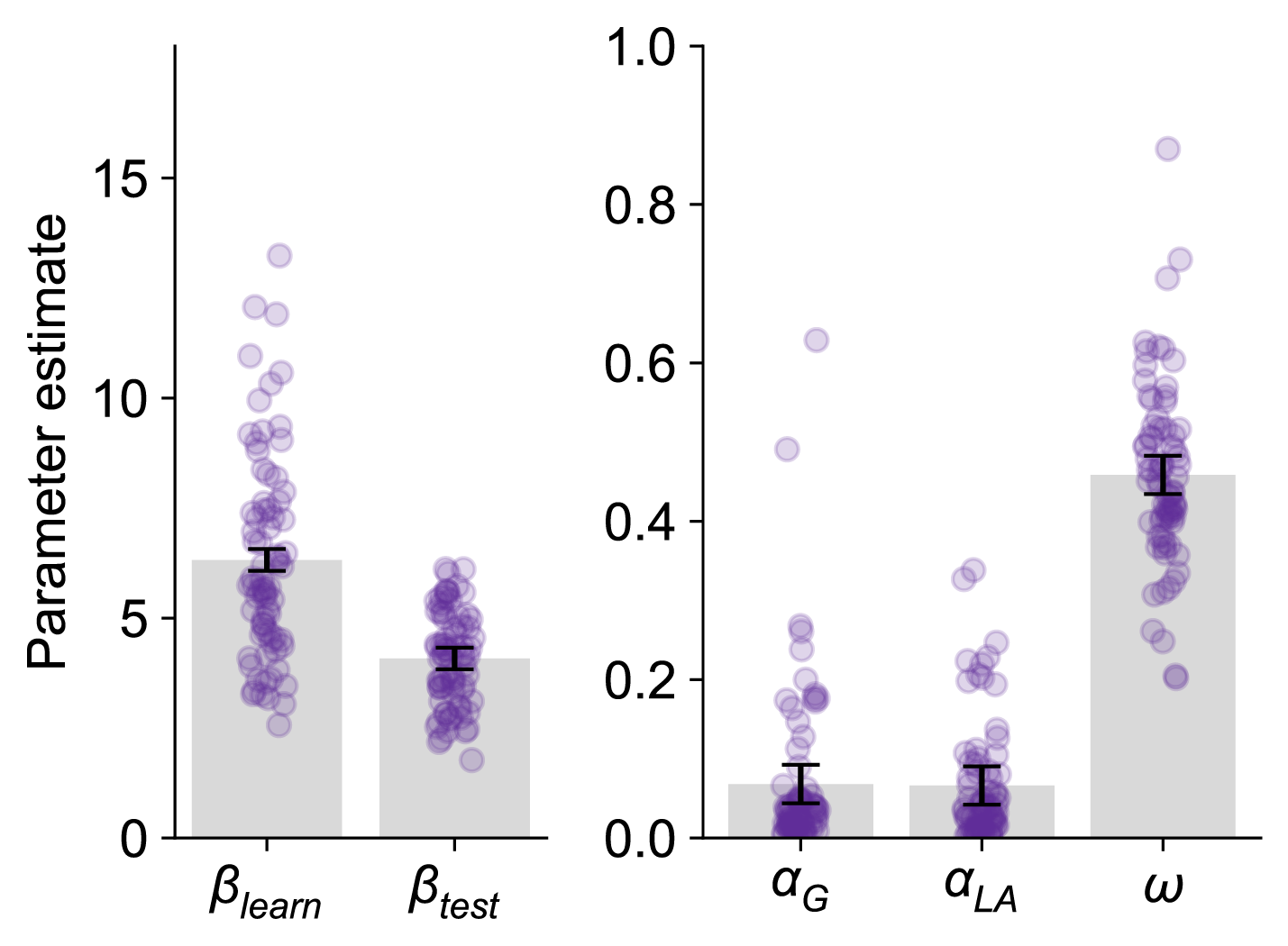

Supplement: S10 Fig — Computational modeling scripts used to produce this figure are available at https://osf.io/sfnc9/. (TIF) [file pbio.3002201.s011.tif]

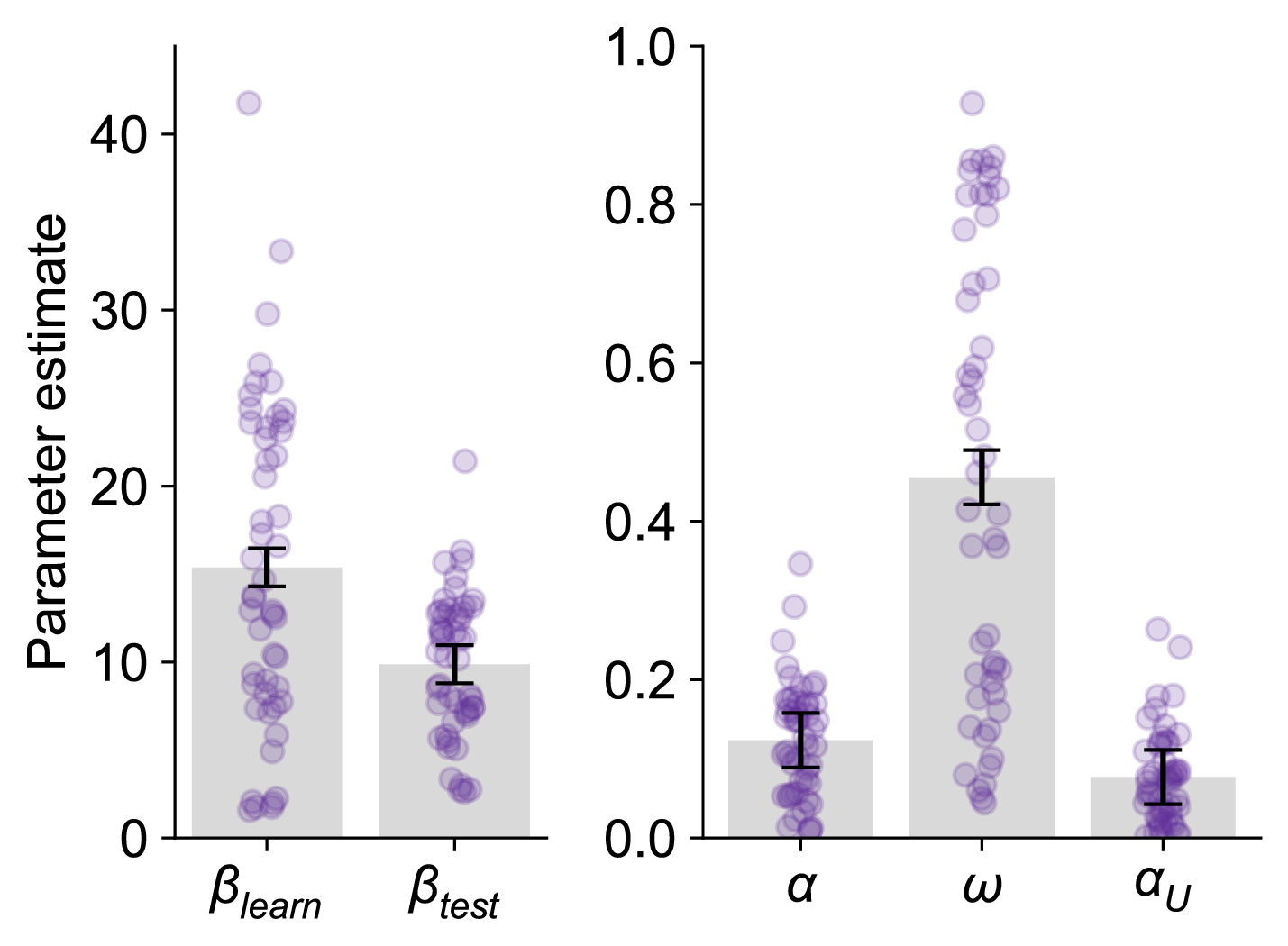

Supplement: S11 Fig — Computational modeling scripts used to produce this figure are available at https://osf.io/sfnc9/. (TIF) [file pbio.3002201.s012.tif]

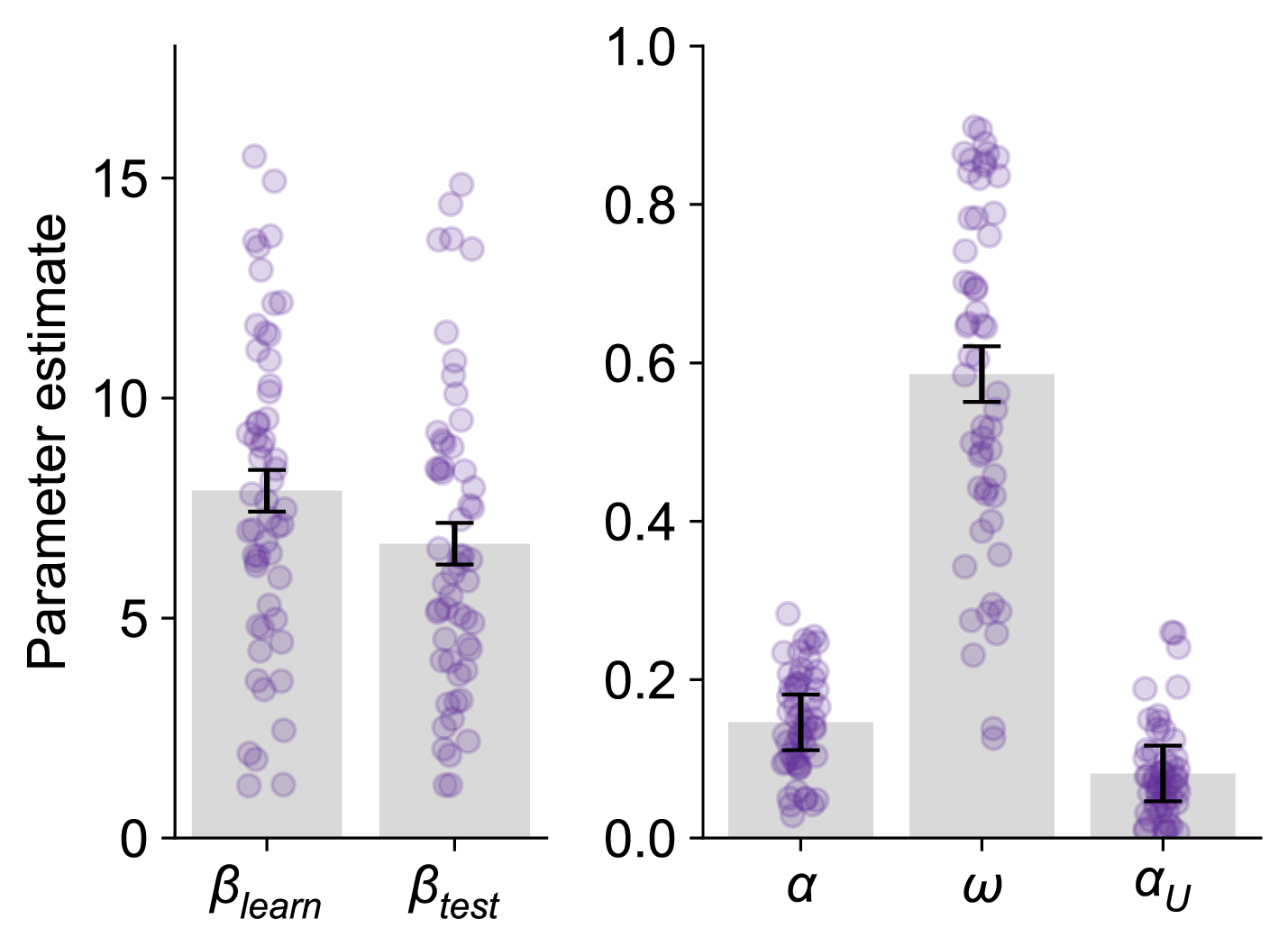

Supplement: S12 Fig — Computational modeling scripts used to produce this figure are available at https://osf.io/sfnc9/. (TIF) [file pbio.3002201.s013.tif]

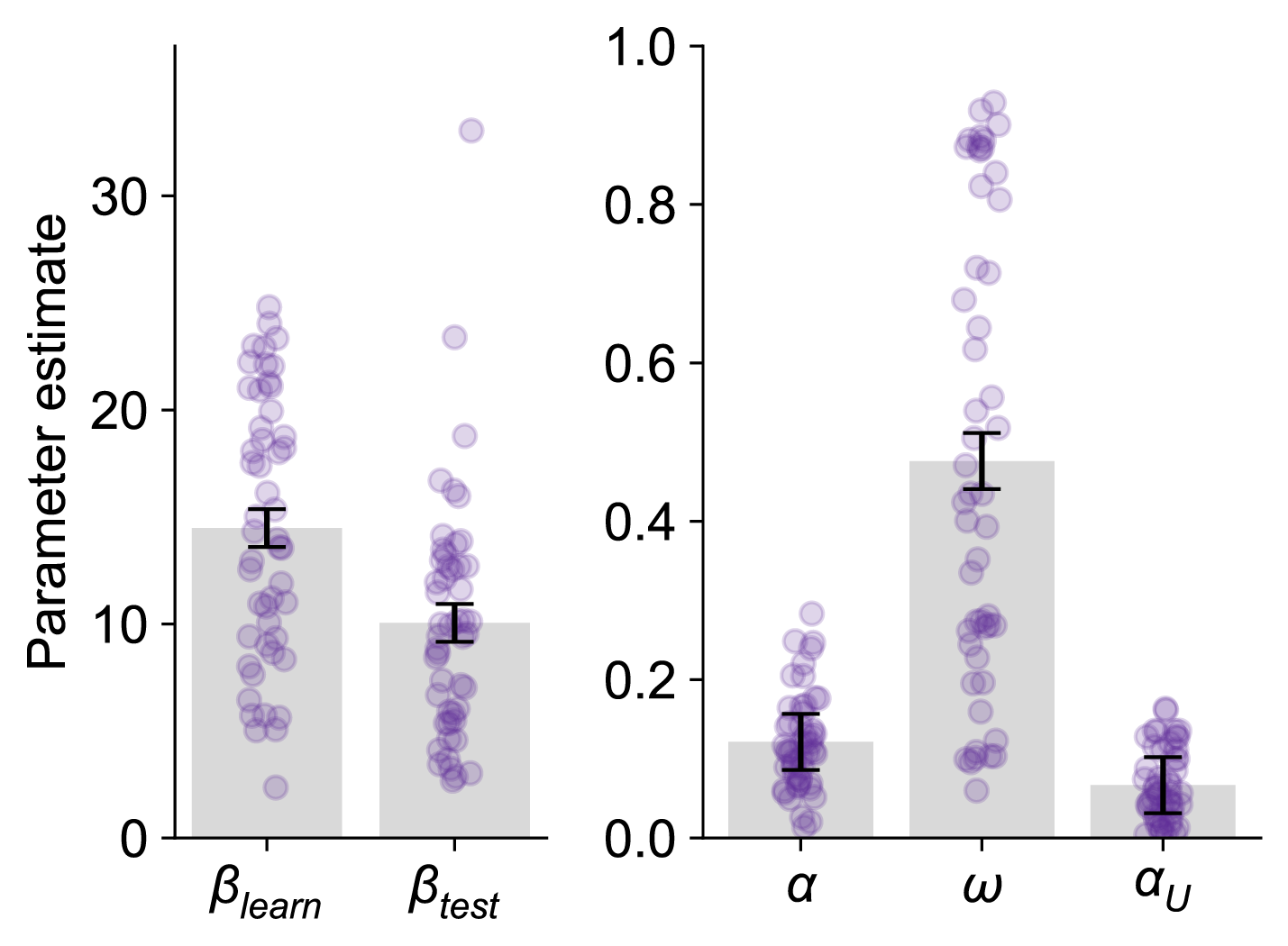

Supplement: S13 Fig — Computational modeling scripts used to produce this figure are available at https://osf.io/sfnc9/. (TIF) [file pbio.3002201.s014.tif]

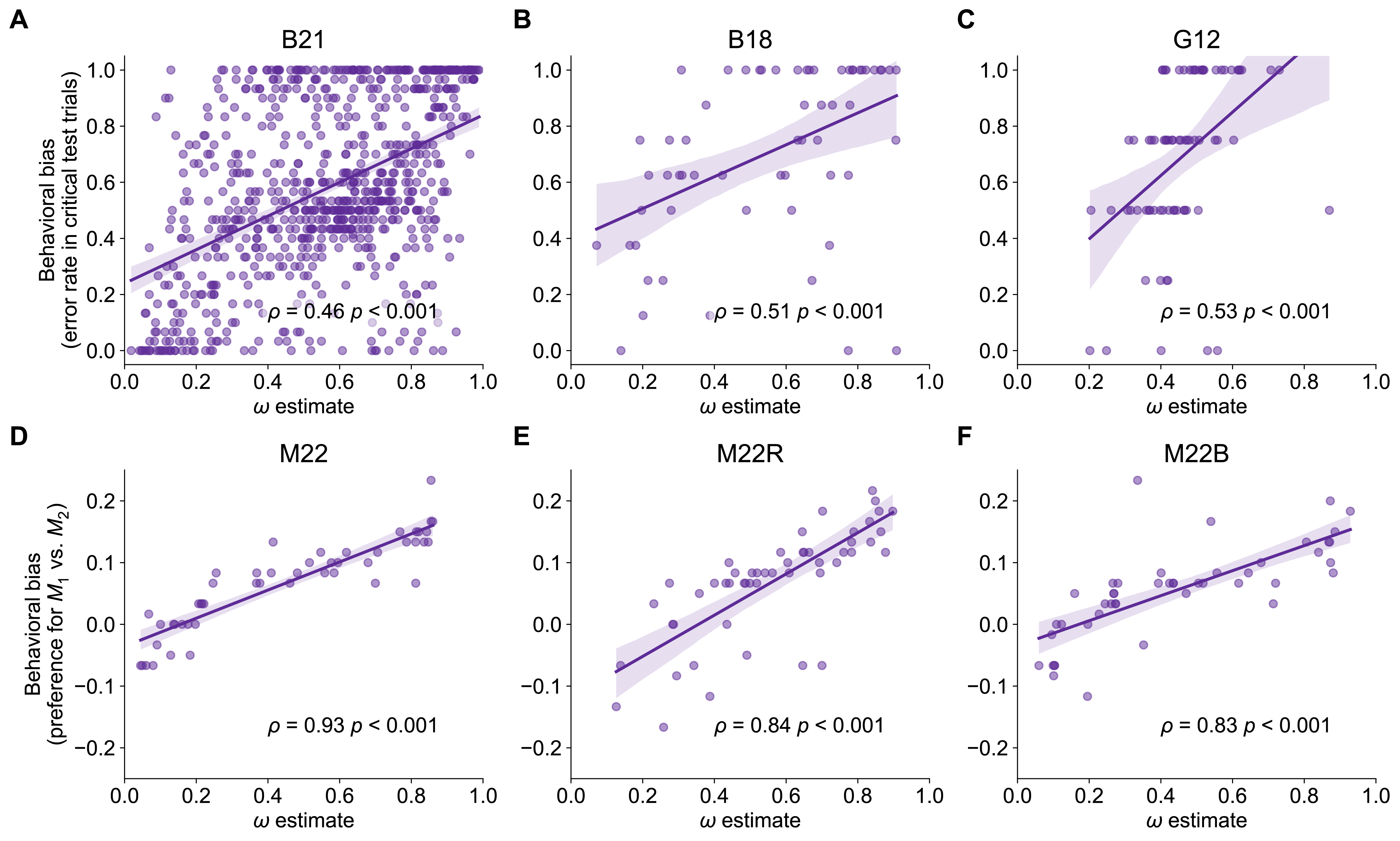

Supplement: S14 Fig — (A) In B21, ωlearn correlates with the error rate in context 8 (choosing a bandit with EV = 0.75 vs. one with EV = 2.5. (B) In B18, ω correlates with the average error rate when choosing between a bandit with EV = 0.075 vs. one with EV = 0.25, and between a bandit with EV = −0.025 vs. one with EV = 0.025. (C) In G12, ω correlates with the average error rate when choosing between a bandit with EV = −0.1 vs. one with EV = 0.1, and between a bandit with EV = −0.2 vs. one with EV = 0.2. (D–F) In M22, M22R, and M22B, ω correlates with the difference in choice rates for bandits M1 and M2 (both had EV = 50). Spearman’s ρ is reported for each correlation. All behavioral biases were measured in the test phase. Computational modeling scripts used to produce this figure are available at https://osf.io/sfnc9/. (TIF) [file pbio.3002201.s015.tif]

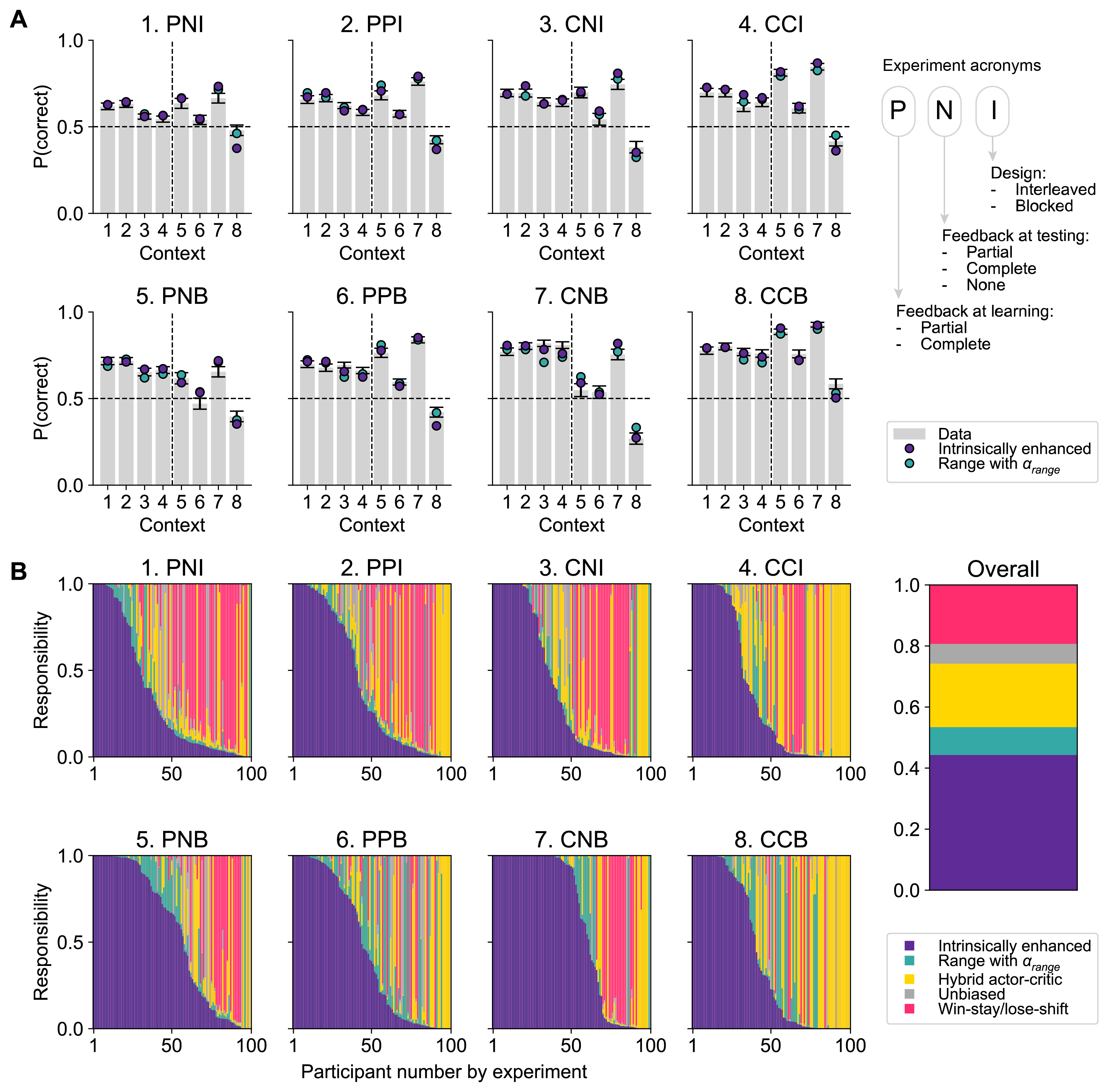

Supplement: S15 Fig — Model validation (A) and comparison (B) by experimental condition in B21 with αrange in the range adaptation model. Here, the range adaptation model was endowed with an αrange parameter, which dynamically updated the minimum and maximum value of each context, as was done in the original study for data set B21 [12]. The addition of this parameter disadvantaged the range model (in teal) compared to the intrinsically enhanced model (in purple). See Fig 2 for caption details. Data underlying this figure are available at https://github.com/hrl-team/range/. Computational modeling scripts used for the illustrated results are available at https://osf.io/sfnc9/. (TIF) [file pbio.3002201.s016.tif]
