# Supplementary material for: Intrinsic rewards explain context-sensitive valuation in reinforcement learning
Source: PLoS Biol. 2023 Jul 17;21(7):e3002201. doi: 10.1371/journal.pbio.3002201 (PMC10374061; doi:10.1371/journal.pbio.3002201)
Supplement: S1 Text — (PDF) [file pbio.3002201.s017.pdf]

For experiment M22R, 55 participants (age M = 20.17 ± 0.19, age range = 18-24, 74% female) were recruited from the university's pool of participants via Sona (`https://www.sona-systems.com`), completed the experiment from their own devices, and were compensated with course credits for their time (around 20 minutes, on average). Participants followed the same protocol as those in the main study. The number of participants exceeded the preregistered aim of 50 due to overestimating the participants' drop-out rate. This difference in N was the only deviation from the preregistered analysis plan.

All behavioral and modeling results from the main study were successfully replicated. Participants performed above chance in both the learning (M = 0.88 ± 0.02; t(54) = 20.69, $p<0.001$; S3 Fig) and the test phase of the experiment (M = 0.87 ± 0.02; t(54) = 23.01, $p<0.001$). They chose option M_1 (mean choice rate across all trials in the test phase: 0.58 ± 0.02) more often than option M_2 (0.33 ± 0.03; t(54) = 6.1, $p<0.001$; S2A-B Fig). When the two options were pitted against each other, participants selected M_1 significantly more often than chance (i.e., 0.50; M = 0.87 ± 0.02; t(54) = 23.01, $p<0.001$; S2C Fig). Performance in the test phase was better for trials in which the M_1 option was pitted against either low option (M = 0.90 ± 0.03) than when M_2 was pitted against either low option (M = 0.65 ± 0.04; t(54) = 5.28, $p<0.001$; S2C-D Fig). By contrast, performance in the test phase was better for trials in which the M_2 option was pitted against either high option (M = 0.93 ± 0.02) than when M_1 was pitted against either high option (M = 0.85 ± 0.03; t(54) = -2.59, p = 0.012; S2C-D Fig). Participants' explicit evaluations were higher for M_1 (61.33 ± 2.45) than M_2 (M = 39.62 ± 2.38; t(54) = 5.97, $p<0.001$; S2E-F Fig). Besides capturing behavior better than range^z, the intrinsically enhanced model was the most frequently expressed (protected exceedance probability = 0.98 versus 0.02 for the range^z model) and had the highest responsibility across participants (intrinsically enhanced = 0.63, range adaptation = 0.35, win-stay/lose-shift = 0.02; S2G Fig). The intrinsically enhanced model's ω parameter (M = 0.59 ± 0.03) was significantly correlated with the difference in choice rates for M_1 vs. M_2 in the test phase (Spearman's $\rho = 0.84$, $p < 0.001$; S14E Fig).
