# Supplementary material for: Intrinsic rewards explain context-sensitive valuation in reinforcement learning
Source: PLoS Biol. 2023 Jul 17;21(7):e3002201. doi: 10.1371/journal.pbio.3002201 (PMC10374061; doi:10.1371/journal.pbio.3002201)
Supplement: S2 Text — (PDF) [file pbio.3002201.s018.pdf]

For experiment M22B, 50 participants (age M = 31.28 ± 0.85, age range = 18-40, 26% female) were recruited via Prolific (`https://www.prolific.co`) with the same modalities as in the main study (M22). Participants performed the same task as described before, except that, for each context, all three bandits and outcomes were always displayed (S4 Fig). However, on some trials – following the same dynamics as in the main experiment (Fig 6) – only two of the options could be selected, and one of them was blocked. Unavailable options were unresponsive and displayed as more opaque than available ones. Compared to the main study, this control encouraged participants to view choices with only two available options as part of the same context as other choices employing the same stimuli.

Once again, we confirmed the results of our initial study. Participants' performance was above chance in both the learning (M = 0.94 ± 0.01; t(49) = 33.4, p<0.001; S6 Fig) and the test phase of the experiment (M = 0.91 ± 0.01; t(49) = 28.48, p<0.001). Option M_1 (mean choice rate across all trials in the test phase: 0.55 ± 0.02) was chosen more often than option M_2 (0.35 ± 0.03; t(49) = 5.62, p<0.001; S5A-B Fig). When M_1 was pitted against M_2, participants chose the former more often than predicted by chance (i.e., 0.50; M = 0.75 ± 0.05; t(49) = 5.16, p<0.001; S5C Fig). Participants performed better in test trials where the M_1 option was pitted against either low option (M = 0.92 ± 0.02) than when M_2 was pitted against either low option (M = 0.72 ± 0.05; t(49) = 4.53, p<0.001; S5C-D Fig). By contrast, they performed better in test trials where the M_2 option was pitted against either high option (M = 0.92 ± 0.02) than when M_1 was pitted against either high option (M = 0.97 ± 0.01; t(49) = -2.47, p = 0.017; S5C-D Fig). Participants' explicit ratings were higher for M_1 (55.84 ± 1.43) than M_2 (M = 41.69 ± 2.30; t(49) = 4.76, p<0.001; S5E-F Fig). The intrinsically enhanced model captured behavior better than range^z and was the most frequently expressed (protected exceedance probability = 0.98 versus 0.02 for the range^z model) and had the highest responsibility across participants (intrinsically enhanced = 0.63, range adaptation = 0.35, win-stay/lose-shift = 0.02; S5G Fig). The intrinsically enhanced model's ω parameter (M = 0.48 ± 0.04) was significantly correlated with the difference in choice rates for M_1 vs. M_2 in the test phase (Spearman's $\rho = 0.83$, $p < 0.001$; S14F Fig).
