# Supplementary material for: Intrinsic rewards explain context-sensitive valuation in reinforcement learning
Source: PLoS Biol. 2023 Jul 17;21(7):e3002201. doi: 10.1371/journal.pbio.3002201 (PMC10374061; doi:10.1371/journal.pbio.3002201)
Supplement: S3 Text — (PDF) [file pbio.3002201.s019.pdf]

A preliminary model fitting and comparison using maximum likelihood estimation was conducted on B21 in order to narrow down the space of models under consideration. Additional tested models included models with a single inverse temperature (β) across task phases, models with a single weight for extrinsic vs. intrinsic reward signals (ω, in the intrinsically enhanced model) across task phases, and models with dynamic updating of r_{min} and r_{max} estimates based on a specified learning rate (α_{range}, in range adaptation models). In general, these models were less able to capture participants' behavior. The α_{range} was particularly problematic, as it was difficult to recover and it disadvantaged range models by adding undue complexity (S15 Fig). For data sets B18 and G12, models with a shared learning rate across positive and negative stimuli were considered, but eventually excluded due to their reduced ability to match participants' behavior.
