# Supplementary material for: Intrinsic rewards explain context-sensitive valuation in reinforcement learning
Source: PLoS Biol. 2023 Jul 17;21(7):e3002201. doi: 10.1371/journal.pbio.3002201 (PMC10374061; doi:10.1371/journal.pbio.3002201)
Supplement: Attachment — Submitted filename: Context RL Revision 1 Response PLOS Bio.pdf [file pbio.3002201.s020.pdf]

We would like to thank the reviewers for their thorough and thoughtful commentary. We have taken all suggestions into account, and hope the reviewers will agree the paper is now stronger. We report below the reviewers' comments in full for ease of reference, as well as to ensure that each of the points raised has been addressed sufficiently. The reviewers' comments are shown in bold and our response is in normal font. We also report relevant text from the updated main text in blue; here, for the reviewers' convenience, citations are reported in APA style.

## Reviewer 1

**This manuscript from Molinaro and Collins proposes and tests a new computational model for context-sensitive value learning. While context-dependent valuation has largely been examined during decision making (with known option values), recent work has shown that context sensitivity also arises during trial-and-error based reinforcement learning (RL), with much of the literature (Palminteri and others) proposing range adaptation (RA) as a computational explanation. Here, the authors propose an alternative intrinsically enhanced RL model (IEM). in which object valuations are a mixture of classic outcome-based value and an intrinsic reward signal based on goal achievement. Across both previously published datasets and new data, the authors find that the IEM model explains context-sensitive valuation as well as or better than RA.**

**Context-dependent valuation is an important topic in fields like psychology, economics, and computational neuroscience: in contrast to standard normative theories assuming an absolute valuation process, humans show valuation that is context-sensitive and varies with the other current or previously available options. I quite like this paper and its overall approach, proposing a novel, psychologically-motivated, computationally-specified model for reinforcement learning that offers an alternative to range adaptation. The HBI approach to model fitting and comparison seems sophisticated and theoretically well justified, though I do have some questions about how final results (e.g. group parameter estimates) rely on the space of models compared. In addition, i think the authors could clarify a few issues about the form of their model and fit mixture parameter values.**

**Major points**

**(1) Justification for the specific mixture model formulation. The authors do a good job of discussing the general support/evidence for goal-sensitive valuation, but could provide more justification for the specific form of the model they propose. While the idea of a simple mixture model is intuitive, there are some important issues that should be at least addressed. For one, is there a conceptual or empirical reason to expect that values**

**are derived as a (linear) mixture of classic RL-derived estimates and an intrinsic goal signal? Second, is there a particular reason why the intrinsic goal signal is construed as a binary all or nothing signal?**

We thank the reviewer for giving us an opportunity to clarify our model formulation. Both the linearity feature and the binary form of the goal signal are simplifications of a potentially more complex and nuanced model. It is indeed possible that continuous goal signals are integrated non-linearly with absolute outcome values. However, given that our current model captures the data relatively well, we have no reason not to favor the simplest possible formulation – especially since we view our model as a starting point. Moreover, it is not infrequent to come across models of behavior where multiple attributes of the same outcome, such as rewards and effort, costs, or novelty, are linearly combined (e.g., Gläscher et al., 2010; Sharot & Sunstein, 2020). The brain is known to integrate various aspects of an outcome into a single value (Peters & Büchel, 2010; Lak et al., 2014), and even seemingly incommensurable sources of rewards – such as food and monetary outcomes or social cues – are rescaled to similar values during cross-category comparisons (Levy & Glimcher, 2011). As for binary goal signals, similar frameworks have been adopted in the artificial intelligence literature, by which we were partly inspired (Colas et al., 2022). Moreover, some have suggested that valuation may occur by performing binary comparisons, rather than estimating continuous outcomes (Hayes & Wedell, 2022; Stewart et al., 2006), as would be well captured by binary goal-dependent signals. We now make these points clearer in the Discussion (lines 610-620).

> *Lines 610-620*: In addition to the lack of dynamicity, we make the simplifying assumptions that different aspects of outcome valuation are combined linearly and that goal-dependent signals are encoded as binary outcomes. It is not uncommon for researchers to assume that different aspects of an outcome are linearly combined during valuation (Glascher et al., 2010, Sharot & Sunstein, 2020). Succinctly implementing goal-dependent outcomes as binary signals was not only sufficient to capture the data presented here, but also an approach often followed in artificial intelligence research, which our theory was partly inspired by Colas et al., 2022. The brain is known to integrate various aspects of an outcome into a single value (Peters & Büchel, 2010, Lak et al., 2014), and even seemingly incommensurable sources of rewards – such as food and monetary outcomes or social cues – are rescaled to similar values during cross-category comparisons (Levy & Glimcher, 2011).

**One thing that feels missing from the paper is a discussion of what the intrinsic reward signal provides that allows it to capture similar behavior explained by range adaptation: since the IEM model mixes absolute reward and intrinsic reward signals, presumably the**

**intrinsic reward is generating the context-sensitivity. Does this result from the nature of the tasks used here (with learning and test phases), or does this reflect a general relationship between goal attainment and contextual scaling?**

The reviewer is correct that the intrinsic component of the reward signal present in the intrinsically enhanced model is responsible for generating context-sensitivity effects (although, by itself, is insufficient to capture participants' behavior in its entirety – hence the mixed policy). As suggested by the same reviewer below, we correlated model estimates of the ω parameter, where greater values of ω signify a stronger involvement of intrinsically generated rewards in the construction of value signals. Confirming that intrinsic rewards generate context-sensitivity, greater ω estimates were positively correlated with stronger behavioral effects of context-sensitive learning. This is because, by capturing whether a goal has been reached the intrinsically generated signal in the intrinsically enhanced model essentially instantiates a relative policy, wherein the frequency of rewards matters, rather than their absolute outcomes. We expect the model to behave similarly regardless of the task used. That said, in order to observe context-dependence effects, outcomes need to be experienced in separate, fixed contexts (see Juechems et al., 2022). We would also like to note that, while context-sensitivity effects are *evident* in the test phase of each of the analyzed tasks (where all, or critical combinations of bandits are assessed), context sensitivity is *acquired* in the learning phase. We now clarify in the Discussion that context-sensitivity is achieved by defining goals-dependent signals in relation to the best possible outcome (lines 497-499).

> *Lines 497-499:* Here, context-sensitivity is achieved by defining goal-dependent signals in relation to the best possible outcome.

**(2) Consistency between replicating experimental data and model fitting metrics. There are several instances where the authors state that the IEM model better fits the behavioral data but the model comparison favors the RA model, for example the B18 dataset (IEM more closely matches data but RA is the most frequently expressed model) and the M22 replication dataset (text_s1). Can the authors clarify why there is this discrepancy where the group level parameters seem to favor the IEM even though across individuals the IEM is not the winning model?**

We thank the reviewer for this opportunity to explain inconsistencies between a given model's ability to replicate subjects' behavior and its responsibility at the individual level. To clarify, we believe that two factors contribute to such discrepancies. First, for data sets B21, B18, and G12, the number of parameters is greater for model IEM compared to range adaptation. While the HBI fitting procedure has been shown to prevent overpenalizing complex models (Piray et al., 2019), it is still possible that the additional complexity

inherent in the IEM was not sufficient to compensate for its better ability to capture behavior. Additionally, while for all studies we were mainly interested in behavior during the testing phase, models were fit on both the learning and testing phases. Therefore, it is possible that models competing with IEM were marginally, and occasionally, better at describing participants' behavioral fluctuations in the learning phase. Regardless of its relative position in model comparison, for a model to be considered a valid account of participants' behavior, it must be able to replicate the key patterns one is attempting to model (Palminteri et al., 2017). We now provide a succinct explanation for the discrepancies noted by the reviewer in the main text (lines 254-257).

> *Lines* 254-257: Although the HBI pipeline has been shown to avoid overpenalizing complex models (Piray et al., 2019), it is still possible that the additional complexity of the intrinsically enhanced model was not sufficient to compensate for its better ability to capture behavior.

All the factors mentioned above notwithstanding, for the M22R dataset (Text S1), we – like the reviewer – were puzzled by the inconsistency between the models' ability to replicate behavioral data and their ranking among candidate models. This prompted us to re-examine this data set more closely. Since submitting our manuscript, we have identified a bug in our data preprocessing pipeline, which affected data sets M22 and M22R. Specifically, in a small number of trials, there was a mismatch between the order of outcomes 1, 2, and 3 (appearing on the left, middle, and right, respectively) in the data frame and the actual order in which they appeared (which was retrievable from other columns in the raw file). Upon correcting this issue and updating the data sets, IEM resulted as the winning model in M22R as well as M22, consistent with behavior simulation results. The corrected data frames have now been uploaded to the previously indicated repository (https://osf.io/2sczg/?view_only=892ad07170284914a35a485f40320aa9), and the results have been corrected in the new submission (lines 461-464, Fig 8, Text S1, and Fig S2).

> *Lines* 461-464: The intrinsically enhanced model was the most frequently expressed (protected exceedance probability = 1) and had the highest responsibility across participants (intrinsically enhanced = 0.83, range adaptation = 0.09, win-stay/lose-shift = 0.08; Fig 8G).

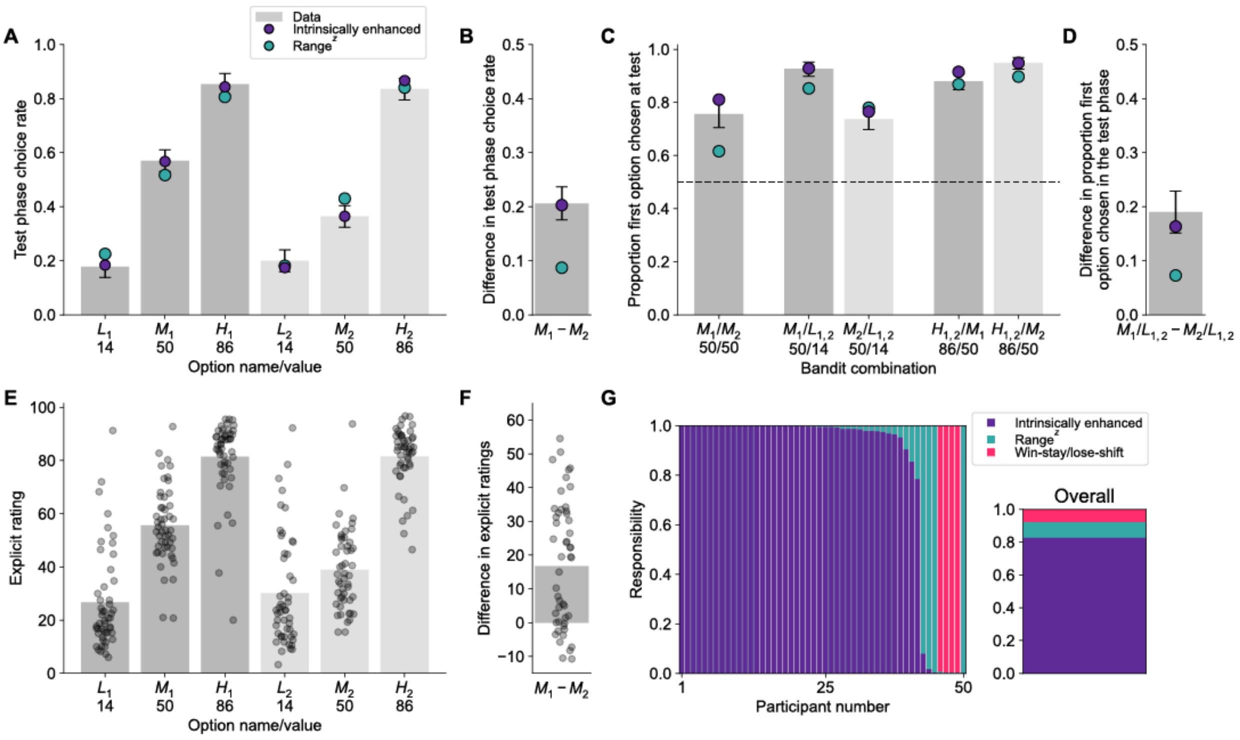
A
B
C
D
E
F
G
Data
Intrinsically enhanced
Range$^z$
Test phase choice rate
Option name/value
$L_1$ 14
$M_1$ 50
$H_1$ 86
$L_2$ 14
$M_2$ 50
$H_2$ 86
Difference in test phase choice rate
$M_1 - M_2$
Proportion first option chosen at test
$M_1/M_2$ 50/50
$M_1/L_{1,2}$ 50/14
$M_2/L_{1,2}$ 50/14
$H_{1,2}/M_1$ 86/50
$H_{1,2}/M_2$ 86/50
Bandit combination
Difference in proportion first option chosen in the test phase
$M_1/L_{1,2} - M_2/L_{1,2}$
Explicit rating
Difference in explicit ratings
Responsibility
Participant number
Intrinsically enhanced
Range$^z$
Win-stay/lose-shift
Overall

*Fig 8*

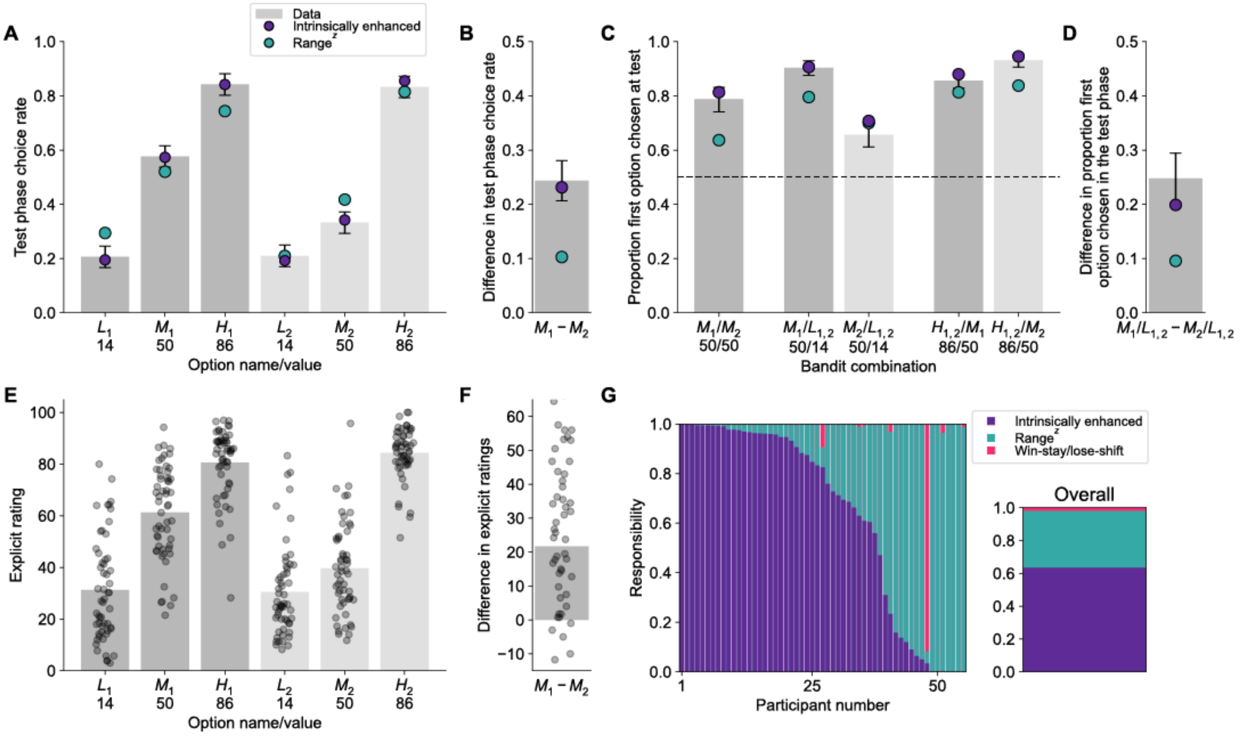
A
B
C
D
E
F
G
Data
Intrinsically enhanced
Range$^z$
Test phase choice rate
Option name/value
$L_1$ 14
$M_1$ 50
$H_1$ 86
$L_2$ 14
$M_2$ 50
$H_2$ 86
Difference in test phase choice rate
$M_1 - M_2$
Proportion first option chosen at test
$M_1/M_2$ 50/50
$M_1/L_{1,2}$ 50/14
$M_2/L_{1,2}$ 50/14
$H_{1,2}/M_1$ 86/50
$H_{1,2}/M_2$ 86/50
Bandit combination
Difference in proportion first option chosen in the test phase
$M_1/L_{1,2} - M_2/L_{1,2}$
Explicit rating
Difference in explicit ratings
Responsibility
Participant number
Intrinsically enhanced
Range$^z$
Win-stay/lose-shift
Overall

*Fig S2*

**(3) Presenting model parameters. The crucial parameter in the IEM model is the mixture parameter omega, and I think it would be informative if the authors presented best fit model parameters (either group level, or average population) across the different datasets. The authors briefly discuss how omega varies across different task variants in the B21 dataset (section 1.3.4), but it would be useful to see how omega varies across datasets. I realize that some of this information is plotted in the SI (e.g. Fig S6), but given its central role in the model I'd like to see information about omega in the main text.**

Following the reviewer's suggestion, we now report the mean and standard deviation for ω alongside the results for each data set (lines 169-172 (B21), 241-246 (B18), 295-299 (G12), 471-474 (M22), Text S1 (M22R), and Text S2 (M22B – a new control experiment which we detail below)). These manuscript modifications are reported in response to the next, related point.

**Related points: Have the authors examined whether the variability in omega (as seen in fFg s5) correlates with behavioral measures of context-dependence (or other general behavioral performance features) across individuals?**

The reviewer's suggestion presents an opportunity to perform an interesting sanity check. All else being equal, the higher the ω parameter, the stronger the behavioral signatures of context dependence should be. We correlated ω with the key behavioral signature of context-sensitive learning, and found significant, positive correlations in each data set. These are now reported in Fig S14 as well as in the main text (lines 169-172 (B21), 241-246 (B18), 295-299 (G12), 471-474 (M22), Text S1 (M22R), and Text S2 (M22B)). The following behavioral metrics were used: error rate in context 8 (choosing a bandit with EV = 0.75 vs. one with EV = 2.5; Fig S14A) for B21, average error rate when choosing between a bandit with EV = 0.075 vs. one with EV = 0.25, and between a bandit with EV = -0.025 vs. one with EV = 0.025; Fig S14B) for B18, average error rate when choosing between a bandit with EV = -0.1 vs. one with EV = 0.1, and between a bandit with EV = -0.2 vs. one with EV = 0.2; Fig S14C) for G12, and difference in choice rates for bandits $M_1$ and $M_2$ (both had EV = 50; Fig S14D-F) for M22, M22R, and M22B, respectively.

> *Lines 169-172:* We sought to confirm that the intrinsic reward was instrumental in explaining the behavioral pattern. Indeed, in the intrinsically enhanced model, the ω parameter (M = 0.56 ± 0.01) was significantly correlated with signatures of context-sensitive learning, specifically, the error rate in context 8 (i.e., choosing a bandit with EV = 0.75 vs. one with EV = 2.5; Spearman's $\rho = 0.46$, $p < 0.001$; Fig S14A).

> *Lines 241-246: Moreover, the* intrinsically enhanced model's ω parameter (M = 0.55 ± 0.03) was significantly correlated with key signatures of context-sensitive learning

(i.e., the average error rate when choosing between a bandit with EV = 0.075 vs. one with EV = 0.25 and between a bandit with EV = -0.025 vs. one with EV = 0.025; Spearman's $\rho = 0.51$, $p < 0.001$; Fig S14B), confirming the role of intrinsic reward in explaining context-sensitivity effects.

*Lines* 295-299: Again, the intrinsically enhanced model's $\omega$ parameter (M = 0.46 ± 0.01) was significantly correlated with signatures of context-sensitive learning (i.e., the average error rate when choosing between a bandit with EV = -0.1 vs. one with EV = 0.1, and between a bandit with EV = -0.2 vs. one with EV = 0.2; Spearman's $\rho = 0.53$, $p < 0.001$; Fig S14C).

*Lines* 471-474: The intrinsically enhanced model's\omega parameter (M = 0.46 ± 0.04) was significantly correlated with the difference in choice rates for $M_1$ vs. $M_2$ in the test phase; Spearman's $\rho = 0.93$, $p < 0.001$; Fig S14D).

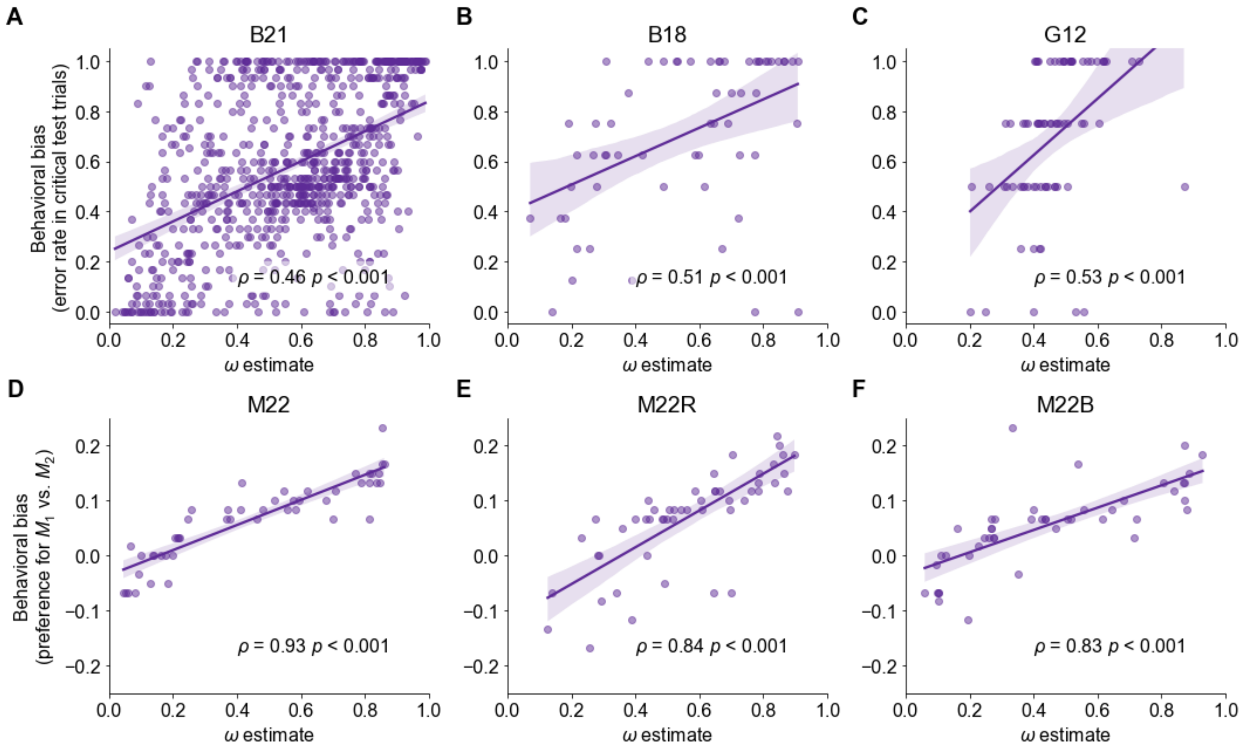


*Fig S14*

**And is there a specific reason that in certain no feedback test phases (PNI, CNI, etc.) the omegas cluster at 0.33? I presume this was set up as such, but it would be good to see it stated.**

As the reviewer correctly noted, there are some experiments of B21 for which $\omega_{test}$ values cluster at around 0.33. This is because $\omega_{test}$ values can only be fit in experiments for which feedback is provided in the test phase. Accordingly, $\omega_{test}$ is absent from all other analyzed data sets, none of which included experiments with feedback at testing. In cases of B21 where feedback at testing was unavailable, $\omega_{test}$ was irrelevant and therefore set to the HBI prior, as was noted in the caption for Fig S8.

> *Fig S8 legend:* Best fit parameters for the intrinsically enhanced model in B21. Note that $\alpha_U$ could only be fit in experiments with counterfactual feedback, and $\omega_{tesst}$ could only be fit in experiments with counterfactual feedback at testing, and are therefore left at the initial prior. [...]

**(4) Clarification of model fitting and simulation results. As I understand it, the authors used hierarchical Bayesian inference (HBI) to fit the different models, which estimates model parameters with population distributions while simultaneously accounting for the assignment of different models to different individuals. First, can the authors clarify what they mean when they simulate data from different models (e.g. Fig 2A, Fig 3 left panel, Fig 4 left panel): are these the result of group level model parameters? And do these group level parameter estimates for a given model include an effect of all individuals, even though some (or many) of those individuals have other models with the highest responsibility? I believe that HBI weights the group level estimates accordingly, but it would be good (A) for this information to be specified in the Methods or Results somewhere, and (B) for the figure legends to be more clear about what is being plotted. Second, at a more conceptual level, does this approach mean that group level parameter estimates will depend at least partially on what companion models are included in the fitting process?**

The HBI fitting pipeline involves two steps: first, the best-fitting parameters are estimated via Laplacian approximation (independent of the model space); second, Laplacian estimates are adjusted based on the likely contribution of each model for each participant's behavior. We now present this explicitly in the Methods (lines 902-907) and in figure legends (Fig 2, Fig 3, Fig 4). Parameters obtained via Laplacian approximation were used for the simulation step as HBI parameters are not always reliable for non-winning models (Piray et al., 2019). The reviewer is correct that group-level parameter estimates depend, at least in part, on which models are included in the fitting process. It is mainly for this reason that we included unbiased and/or win-stay lose/shift models in our model comparison, as adding simple algorithms to the model space has been shown to reduce outliers in group-level parameter estimation (Piray, 2019).

*Lines 902-907:* The HBI fitting pipeline involves two steps: first, the best-fitting parameters are estimated via Laplacian approximation (independent of the model space; MacKay, 2003); second, Laplacian estimates are adjusted based on the likely contribution of each model for each participant's behavior. Parameters obtained via Laplacian approximation were used for the simulation step as HBI parameters are not always reliable for non-winning models (Piray et al., 2019).
*Fig 2 legend:* (A) Model validation by experimental condition and context with parameters extracted through Laplacian estimation (Piray et al., 2019) [...]
*Fig 3 legend:* (A) Model validation by context with parameters extracted through Laplacian estimation [...]
*Fig 4 legend:* (A) Model validation by context with parameters extracted through Laplacian estimation [...]

**Minor points**

**(1) Typo, line 766: "when it r_c,t"**

We thank the reviewer for noticing this typo. The text has now been corrected by removing the "it" in the phrase (line 835).

*Line 835:* Thus, $ir_{c,t}$ was equal to 0 when $r_{c,t}$ was either different from the maximum available reward [...]

**(2) In the Methods description of the hybrid actor-critic model,it's not entirely clear how the parameter h controls the integration of Q-learner and actor-critic values. Perhaps stating the equation would be helpful.**

Following the reviewer's suggestion, we now state an equation for how $h$ regulates the impact of Q-learner values (relative to the hybrid actor-critic weights) on the actor's decisions (equation 19):

$$H_{t+1}(s, c_i) = (1 - h) \cdot W_t(s, c_i) + h \cdot Q_t(s, c_i)$$

where H is the hybrid value upon which the actor's choices are based. Therefore, values of $h$ close to 0 will reduce the model to an actor-critic model, and values of $h$ close to 1 will reduce the model to a Q-learner. In this formulation, we follow Gold et al. (2012).

**(3) For the analysis of the B22 dataset (Figure 5), ti would be helpful if - in addition to the simulated predictions - the authors could show in the figure the empirical behavioral results.**

We agree with the reviewer that including a figure of B22 behavioral results would be helpful. However, data from B22 is (at the time of writing) not publicly available, and we cannot therefore reproduce behavioral results precisely.

**(4) For the ex-ante simulations for B22 and M22 (1.11), the authors draw model parameters from distributions. It would be helpful if the authors stated in teh text a brief rationale for those distributional assumptions.**

The distributions we had selected initially were based on weakly informative priors in accordance with Baribault & Collins (2023). However, we were prompted by the reviewer's comment to reconsider our parameter distribution to improve our clarity and cohesiveness with previous papers (although none of the following edits altered our results). Therefore, we now follow Bavard & Palminteri (2022) for learning rate (α) and inverse temperature (β) parameters. Bavard & Palminteri (2022) do not provide a distribution for the *z* parameter in range. Given that we expect the mean *z* value to vary across experiments, we sampled it from a Gamma(3,3) prior, which has a mean of 1, has a lower bound at 0, and no upper bound. For ω, we sampled parameters from a Beta(2,2) distribution which has a mean of 0.5, a lower bound at 0, and an upper bound at 1. We now state this explicitly in the Methods (lines 883-890) and have updated Fig 5 and Fig 7 accordingly (also shown below).

> *Lines 883-890:* As in Bavard and Palminteri's (2022) work, we sampled the β parameter (inverse temperature) from a Gamma(1.2, 0.2) distribution and the α parameter (learning rate) from a Beta(1.1,1.1) distribution. The *z* parameter for the range adaptation model was drawn from a Gamma(3, 3) distribution (which has a mean of 1, a lower bound at 0 and no upper bound), and the ω parameter (for the intrinsically enhanced model) was drawn from a Beta(2, 2) distribution (which has a mean of 1, a lower bound at 0 and an upper bound at 1).

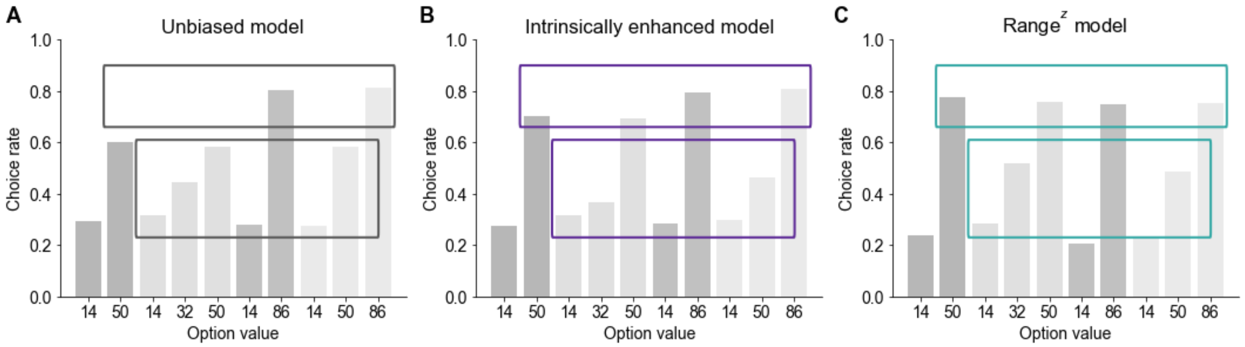


*Fig 5*

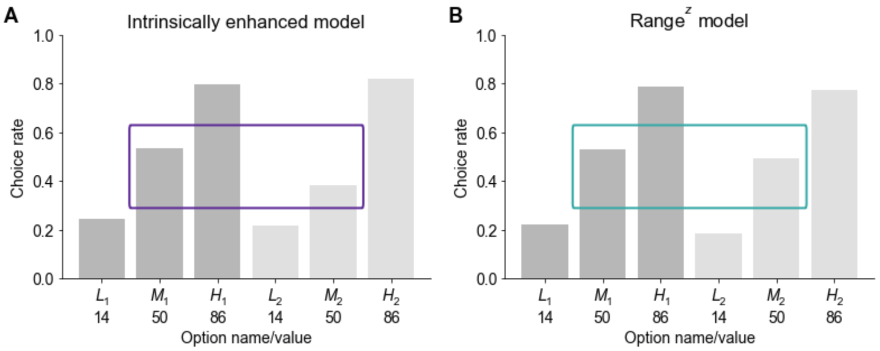


*Fig 7*

**(5) Dissociating the IEM model from range adaptation and reference point centering model. As the authors discuss, the hybrid range adaptation model with reference point centering (from Bavard et al 2018) is mathematically very similar to the IEM, in that they implement a mixture model of absolute and relative reward signals. I actually think the authors could state the advantage of their model a little more strongly: the binary signal used in the Bavard paper is a similar binary signal, but only in specific scenarios where stimuli are offering the same reward with different probabilities of reward (or nothing). Thus, the concept of an intrinsic goal signal is much more general that the very specific from of binary relative value used in Bavard et al.**

We thank the reviewer for their clever observation, and make this aspect clearer in our Results (lines 246-251) and Discussion (lines 649-652).

> *Lines 246-251:* We note that, consistent with our findings, the winning model in (Bavard et al., 2018) was a hybrid between relative and absolute outcome valuation which, in mathematical terms, was equivalent to the intrinsically enhanced model. However, as discussed below (see Discussion), the theory behind the hybrid model presented in (Bavard et al., 2018) aligns more closely with the range adaptation mechanism, and the mathematical overlap only exists for data set B18.
>
> *Lines 649-652:* Crucially, the two models only overlap in specific instances, i.e. tasks in which only binary choices are presented and the same outcomes are associated, though with different probabilities, to options within the same context. This is not the case for the majority of tasks analyzed here.

**(6) For Figure 2, it would really help if the figure had a legend explaining the different letter designations (PNI vs PPI vs CNI). I realize this is in the figure legend, but it would help if it were in the figure image itself.**

Following the reviewer's stylistic suggestion, we have added a legend explaining the different letter designations directly in Fig 2 (illustrated below), which we hope is now more readable.

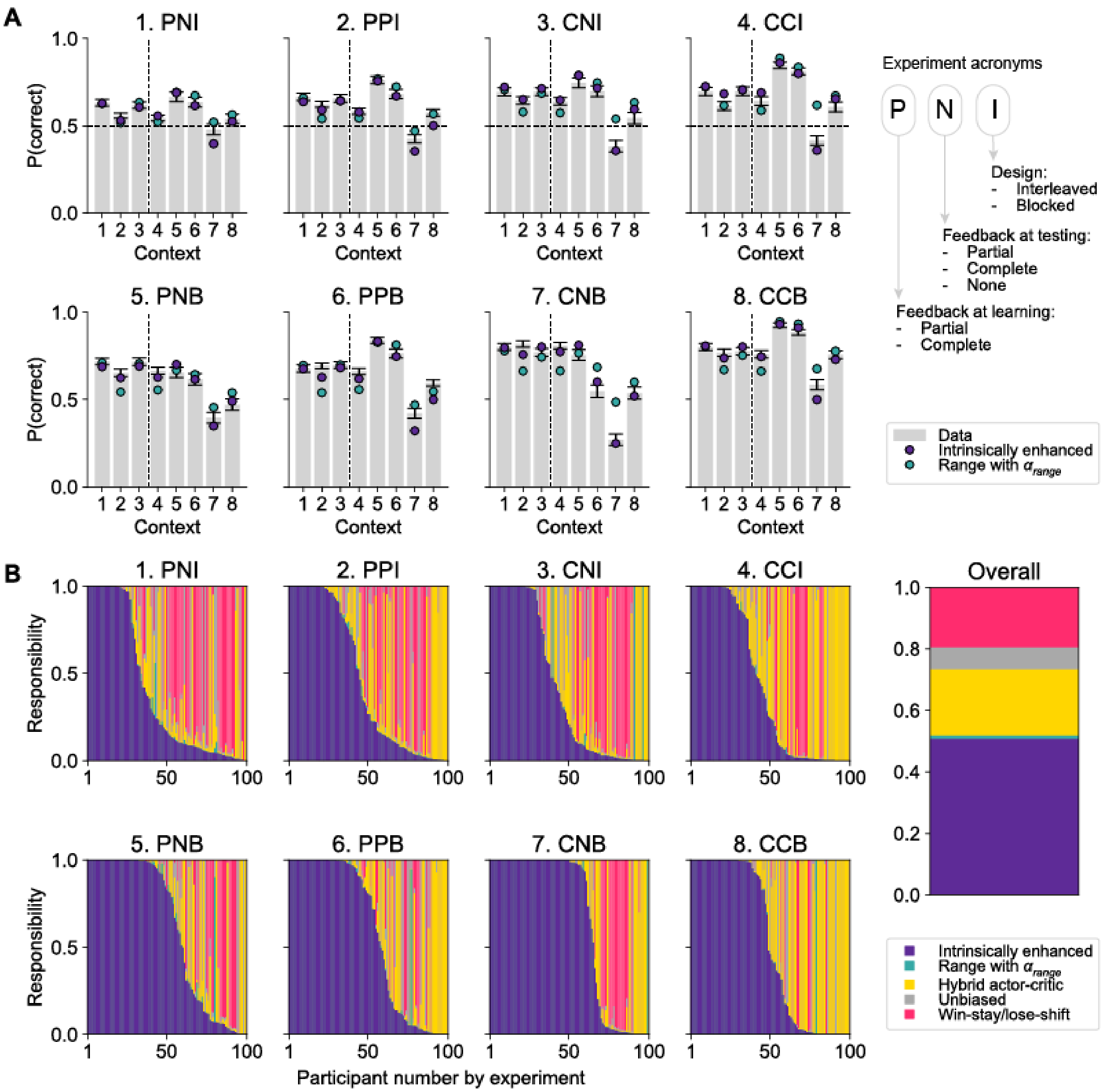


*Fig 2*

## Reviewer 2

**In this manuscript, the authors proposed a new reinforcement learning model enhanced by an intrinsic reward component. Reinforcement learning is a highly influential framework. Although its core computational principles are consensual, how reward is represented is still debated. Recent developments in the fields include range adaptation,**

a phenomenon consisting in rescaling the value function to remain sensitive to the current range of rewards that available in the environment. That is, action outcome would not be reinforcing per se; instead, a normalised outcome (by the reward range) would reinforce the action. As a consequence, people tend to be reinforced to the same extent by the best possible option in a given context, regardless their absolute expected values. Here, the authors proposed a new computational model enhanced by an intrinsic reward component: the outcome value would depend on the agent's goal achievement. The model specifically assumes that participants' goal is to have the best possible performance in a given trial and therefore to choose the best possible option. That is, an outcome reinforces the action to the extent that it corresponds to the best possible outcome available to the participants.

I really enjoyed reading this manuscript for many reasons. I found it to be clearly written. I found the new computational model exciting, as well as the idea to equip the model with an intrinsic reward component. I liked the simulations, the model fit procedure and the model selection metrics (with the use of estimated model frequencies and protected exceedance probabilities) as well as the model recovery analysis. Also, the original experiment has been preregistered.

I however have some concerns which may weaken the results and some suggestions which I hope will help improve the manuscript.

My first concern is related to the results from the authors' original experiment, the only one in the manuscript that can really discriminate between the range adaptation model and the intrinsic reward enhanced model.

1) In the framework of value-based range adaptation, a context is defined to my understanding by the cues that are presented together and is therefore called a "choice context" (see e.g. Palminteri et al., 2015). For example, if a, b and c are always presented together as well as d, e and f, then two contexts are defined. In the authors' original experiment and as per this definition, each trinary (e.g. L1-M1-H1) and each binary choice (e.g. L1-M1) represents a choice context. I guess that's why when three options are used to define a context, they are always presented together in Bavard et al., 2022. A more abstract definition (that the authors seem to embrace) would correspond to the abstract set of stimuli that can be presented together (e.g.L1, M1, H1) even when only two cues of that set are displayed (e.g. M1-H1). I guess that a block design (i.e. L1-M1-H1, M1-H1, L1-H1) would create enough temporal contiguity to create a context corresponding to the latter definition, as would the use of a contextual cue. Yet, in an interleaved design (as in this new experiment), it seems to me that the former definition would apply more (choice context). This is not a purely semantic or conceptual debate, because it has a

**crucial consequence regarding the range adaptation model in this experiment. The key prediction relates to two pairs of cues -L1-M1 in context 1, M2-H2 in context 2. In the transfer phase and according to the intrinsic reward enhanced model, participants should choose more often M1 over M2 because M1 is the best possible option in the given set of option. Yet, range adaptation could (should) occur within a choice context. That is, it would occur for a trinary trial (e.g. L1-M1-H1), as well as within a binary trial (e.g., L1-M1, M2-H2). In such a case, the range adaptation model leads to the exact same prediction as the intrinsic reward enhanced model, weakening considerably (if not cancelling) the evidence supporting the latter.**
**Indeed, M1 would be reinforcing when presented with L1, unlike M2 presented with H2, and therefore M1 would be chosen more often in the transfer phase than M2, as the authors found.**
**Perhaps a cleaner design would be to always present all three options on the screen, defining a clear context 1 & 2. In some trials, participants could only choose between two options (although all three would be displayed) to implement the crucial manipulation from the original experiment. The range adaptation model predicts that the middle options would always be small, while the intrinsic reward enhanced model would predict that the M1 is more selected than M2.**

We thank the reviewer for this insightful comment. We agree that, in many previous studies, the definition of context corresponded to the set of concurrently available options. However, we view this matching pattern as a special case, where context more generally refers to the set of environmental cues that tend to co-occur with a given choice, of which the set of available options is a crucial point, but does not constitute the whole picture. As a real-life example, restaurant A may represent one context, and restaurant B may represent a separate context. The menu of each restaurant is a central piece of what contributes to making the two restaurants separate contexts. However, the occasional unavailability of one of the items on the menu of a restaurant would not necessarily induce the creation of a separate context – although it might shift the representation of what a successful order in the restaurant would look like. We hope the analogy resembles the task used in M22 and M22R enough to be useful. Moreover, we believe that an interleaved trial design should (if anything) *promote* the clustering of two- and three-arm bandits with overlapping stimuli within the same context. This is because an interleaved design would lack the clear division between contexts that a blocked design would come with.

Nonetheless, we agree with the reviewer that potential confounds could be completely eliminated through a control study where all options are always visible, despite the fact that participants can sometimes only choose among two of them (following the same design as the original task we presented). We thus adjusted our previous experiment to avoid this potential confound (M22B; lines 413-425, Text S2, Fig S4). The new results (N=50)

fully confirm our previous behavioral and computational modeling findings (Fig S5-Fig S6, also shown below).

> *Lines 413-425*: We note that, while previous studies defined contexts as the set of available options at the time of a decision (Bavard et al., 2021; Bavard & Palminteri, 2022) here we adopt a more abstract definition of context, which comprises all the options that tend to be presented together – even when only two of them are available (a feature that was absent from previous task designs). Nonetheless, participants might have interpreted choice sets in which only two out of three outcomes were available as separate contexts. With the latter interpretation, range adaptation and intrinsically enhanced models would make the same predictions. To encourage participants to consider the two sets of stimuli as belonging to one context each (even when only two out of three options from a set were available), we designed a control study (M22B; N=50; Text S2) in which, instead of being completely removed from the screen, unavailable options were simply made unresponsive (Fig S4). Therefore, participants could not select unavailable options, but the stimuli and outcomes associated with them were still visible.

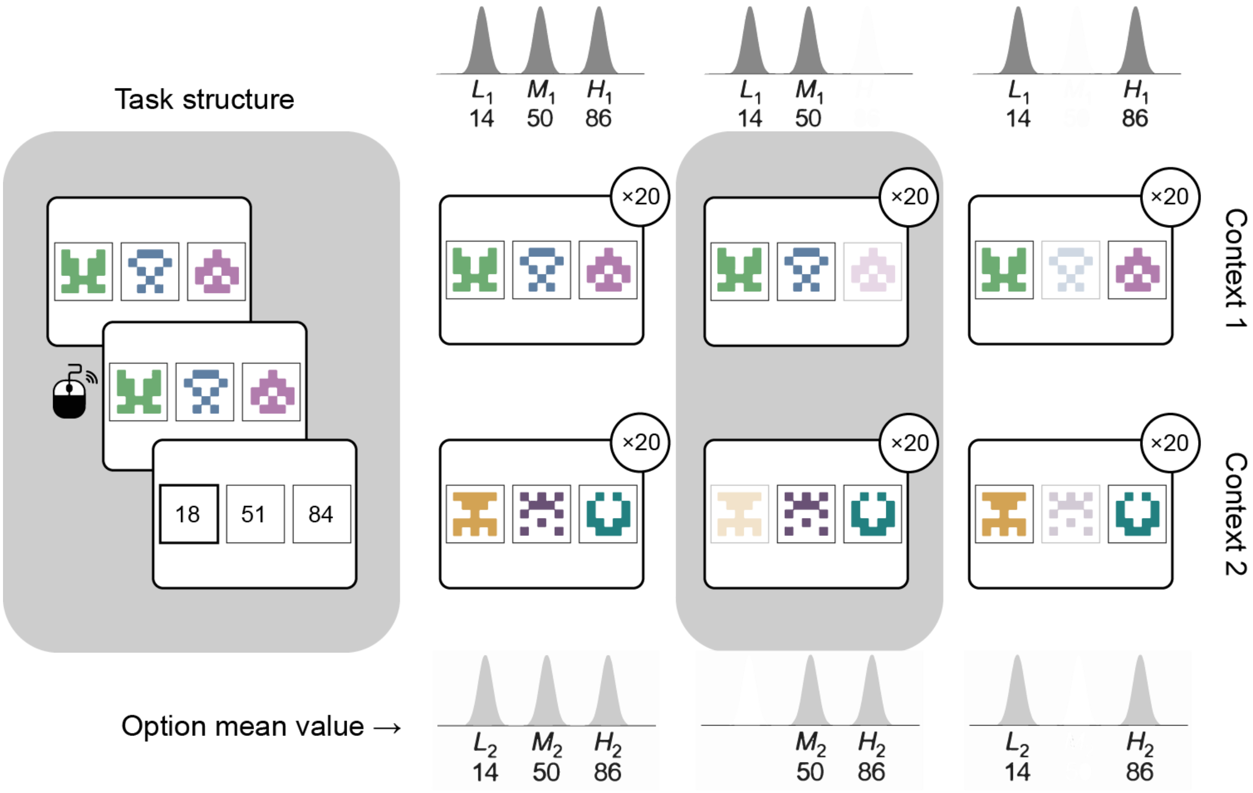


*Fig S4*

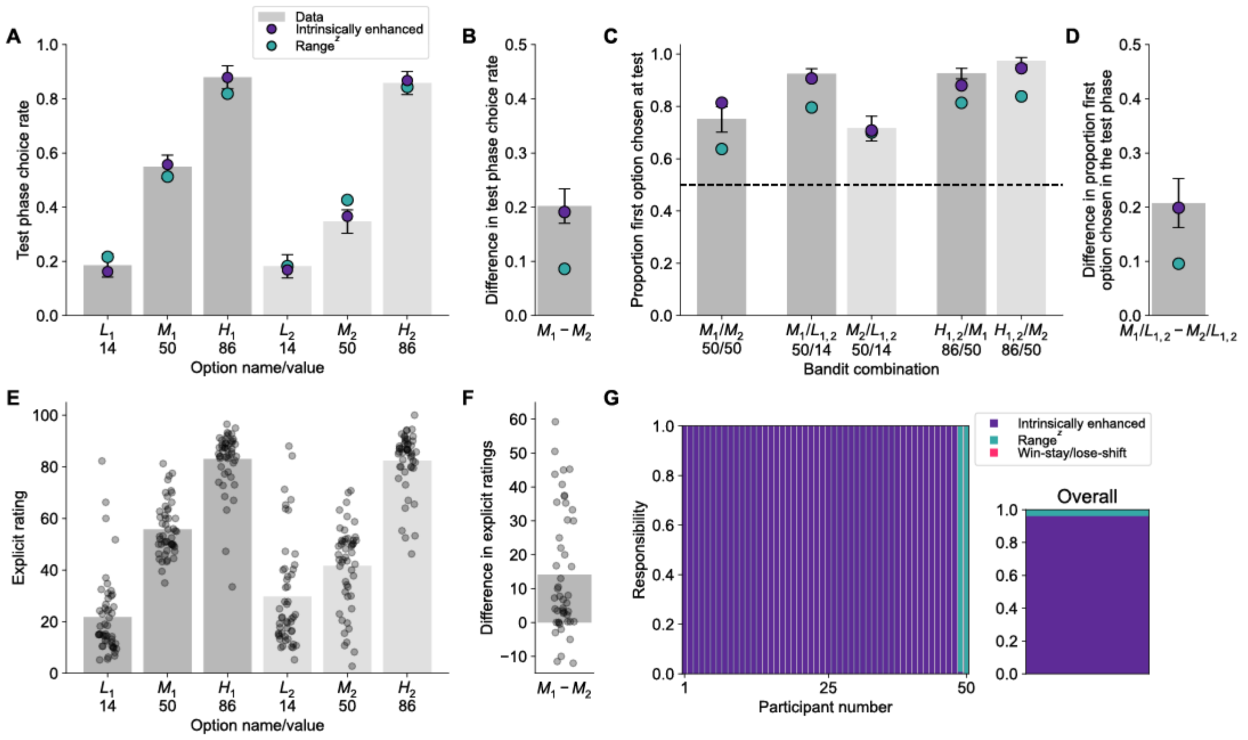


*Fig S5*

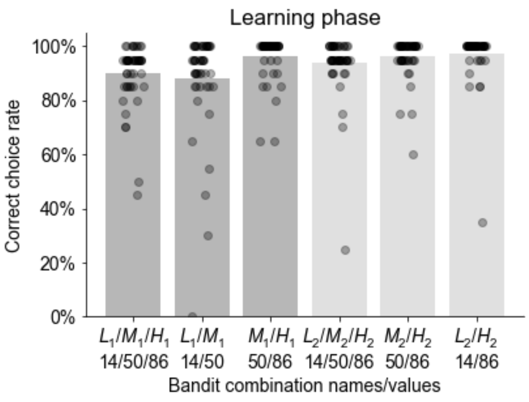


*Fig S6*

**2) Given that the learning rate for the chosen option is usually higher than the learning rate for the unchosen option, I wonder whether the unbiased model would predict the same pattern of results as the intrinsic reward enhanced model: M1 would be updated with a higher learning rate than the unchosen M2 in the M2-H2 pair, leading M1 to have a higher value than M2. I don't think this model is included in any of the reported model space.**

We thank the reviewer for this clever insight. Indeed, the reviewer is correct that higher learning rates for chosen, rather than unchosen options, could lead to biases in favor of $M_1$, as opposed to $M_2$, due to the task design. Our experiment was not designed to distinguish between an unbiased mechanism, which was already excluded by our and other groups' previous analyses. However, the same mechanism described by the reviewer also affects the range *z* model. Indeed, this model does show a slight bias for that $M_1$ option compared to $M_2$ (see for example, Fig 8, teal dot). That said, the magnitude of this effect is much less comparable to that of participants' than the effect we observe for the intrinsically enhanced model (Fig 8, Fig S2, Fig S5 – all reproduced above in response to different comments). As such, while we agree with the reviewer that it is likely that this mechanism contributes to the $M_1 > M_2$ preference, we do not think it can account for the full effect. We now state this explicitly in the Results (lines 467-471).

> *Lines 467-471:* We note that the range adaptation model showed a slight bias for the M1 over the M2 option, likely due to higher learning rates for chosen (compared to unchosen) outcomes and an experimental design where M1 tends to be selected more often – thus acquiring value faster – than M2. Nonetheless, the intrinsically enhanced model matched participants' behavior more closely than range adaption.

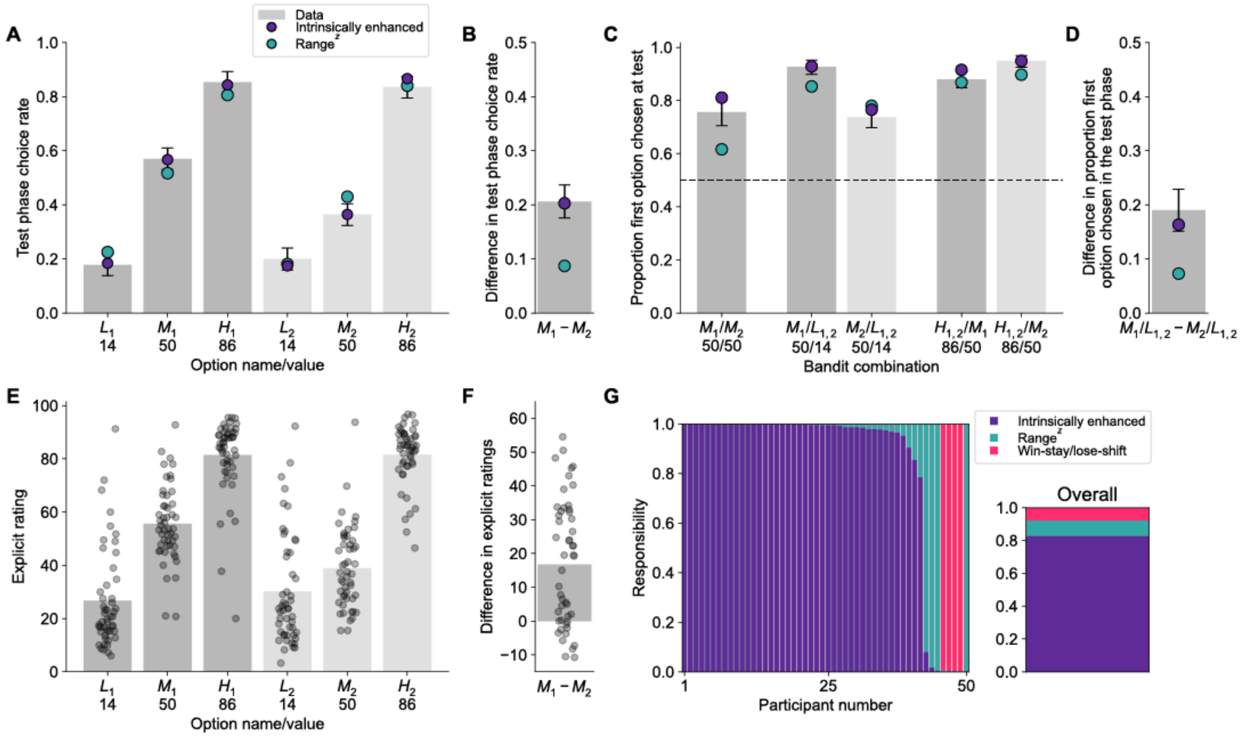


*Fig 8*

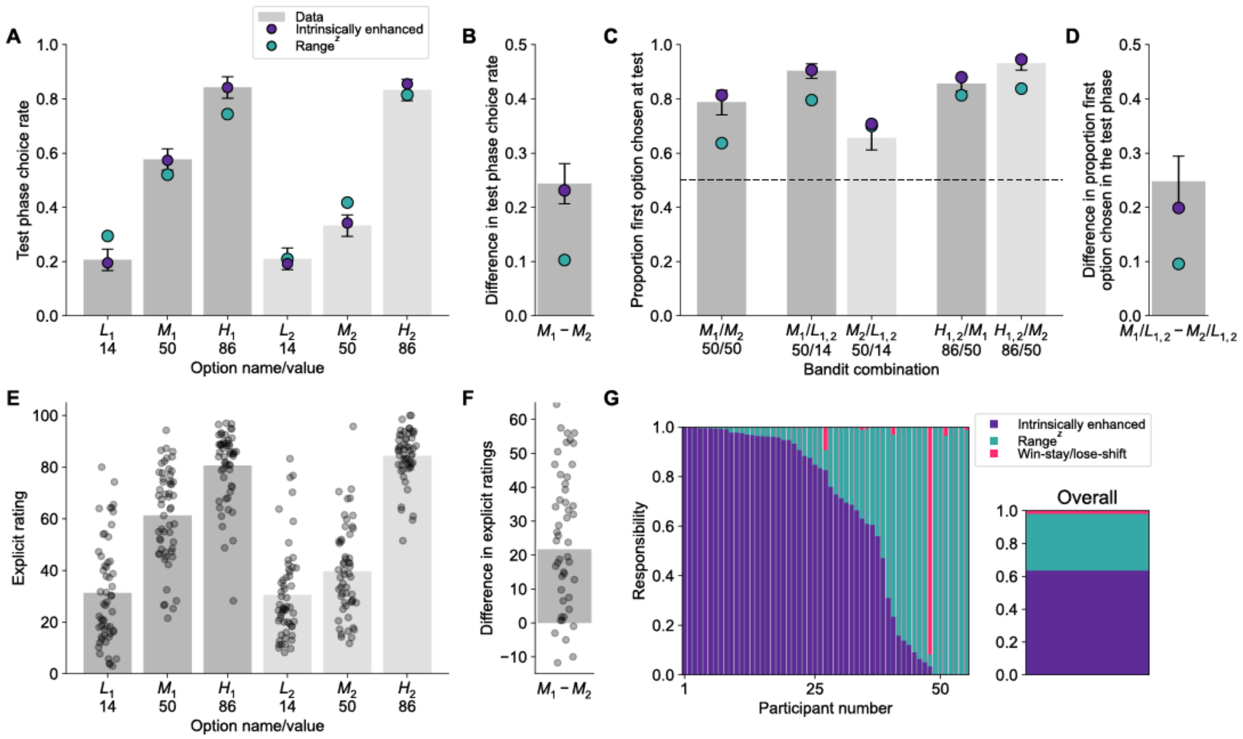

A
B
C
D
E
F
G
Data
Intrinsically enhanced
Range$^z$
Test phase choice rate
Option name/value
Difference in test phase choice rate
$M_1 - M_2$
Proportion first option chosen at test
Bandit combination
Difference in proportion first option chosen in the test phase
$M_1/L_{1,2} - M_2/L_{1,2}$
Explicit rating
Difference in explicit ratings
Responsibility
Participant number
Win-stay/lose-shift
Overall


*Fig S2*

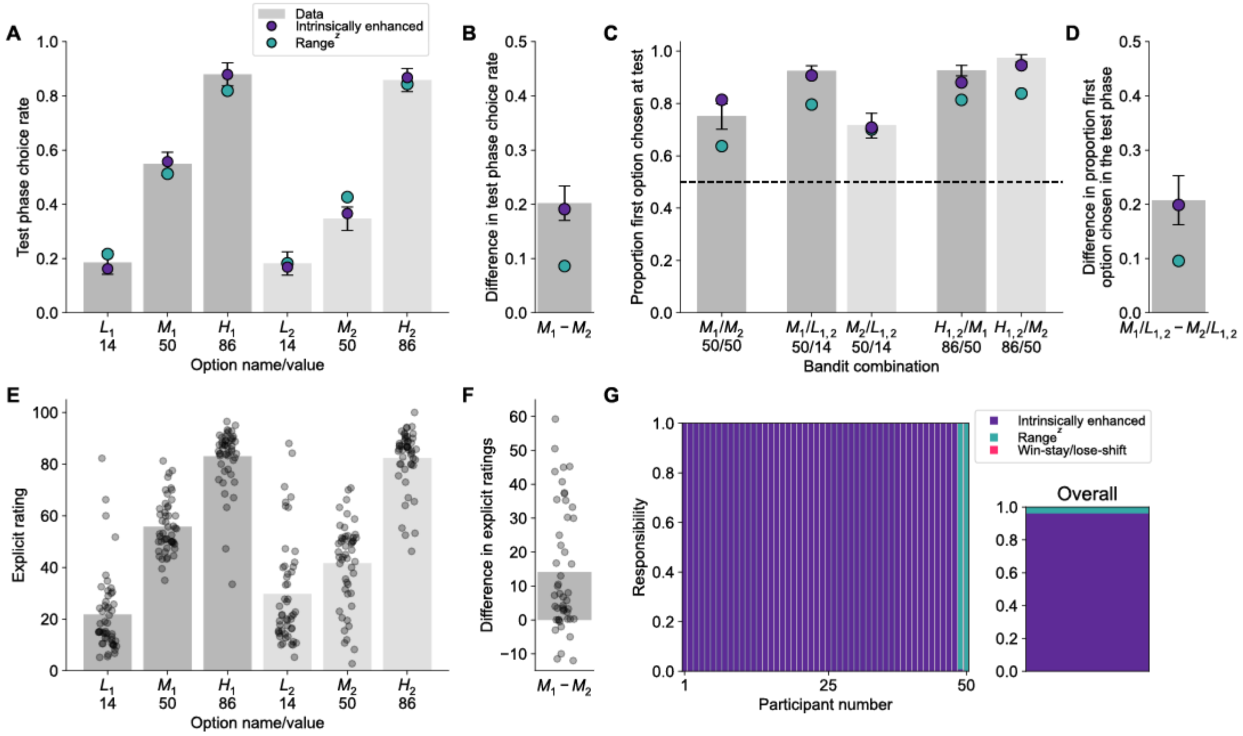

A
B
C
D
E
F
G
Data
Intrinsically enhanced
Range$^z$
Test phase choice rate
Option name/value
Difference in test phase choice rate
$M_1 - M_2$
Proportion first option chosen at test
Bandit combination
Difference in proportion first option chosen in the test phase
$M_1/L_{1,2} - M_2/L_{1,2}$
Explicit rating
Difference in explicit ratings
Responsibility
Participant number
Win-stay/lose-shift
Overall


*Fig S5*

**3) Another major concern I have is related to the comparison between the range adaptation model and the intrinsic reward model. The latter is actually a mixed of the basic model (where the reward is the reward magnitude) and of the intrinsic reward component. A fair comparison would include a mixed of the basic model and of the range adaptation model, as in Bavard et al.,2018 (see equation 2). Alternatively, I suggest to compare a pure intrinsic reward model with the range adaptation model. In this way, the model selection would be much fairer to distinguish between the range adaptation model and of the intrinsic reward model.**

We thank the reviewer for this comment. However, we would like to point out that our candidate model is an intrinsically *enhanced* model, not a purely intrinsic model (although it could capture such behavior by setting $\omega = 1$). Part of the inspiration for such a model came from reasoning that multiple sources of input form reward signals in the human brain (e.g., Kahnt et al., 2011; Juechemes et al., 2018). While an absolute model could well explain people's behavior in some instances, Bavard and colleagues (2018) showed that an absolute model (there equivalent to a purely intrinsic model) was able to explain participants' behavior. The same is true for a purely extrinsic model, as we show in the present article. One of our key contributions, therefore, lies in the very fact that our model presents a mixed policy.

**4) I also suggest to avoid to include too many models in a first model space, to avoid a potential spread of the models in the population that would otherwise be explained by e.g. the range adaptation model. Therefore, I suggest to compare first the range adaptation model with a pure intrinsic reward model, then the mixed range adaptation model and the mixed intrinsic reward model. That would be 2 model spaces with 2 models. Additionally, another model space could include those 4 models and a family model comparison may be performed. Only then a broader model space would be more informative.**

We thank the reviewer for this suggestion. In addition to our answer to the previous comment (comment #3), which we see relevant here too, we would like to note that the inclusion of models that capture simple heuristics (such as the unbiased model and the win-stay/lose-shift model) has been advised as good practice by the developers of the model comparison pipeline we use here, Hierarchical Bayesian Inference (Piray et al., 2019). Moreover, while the reviewer is correct that adding too many models to the model space could detract from the model comparison procedure, we show model recovery above chance (i.e., 20% for experiments with five competing models, and ~33% for experiments with three competing models) in all of our experiments. To stress this latter point, we now show successful model recovery not only for older data sets and M22, but also for M22R

and a new control experiment (referred to as M22B) we ran in response to some of the reviewers' comments (Fig S7).

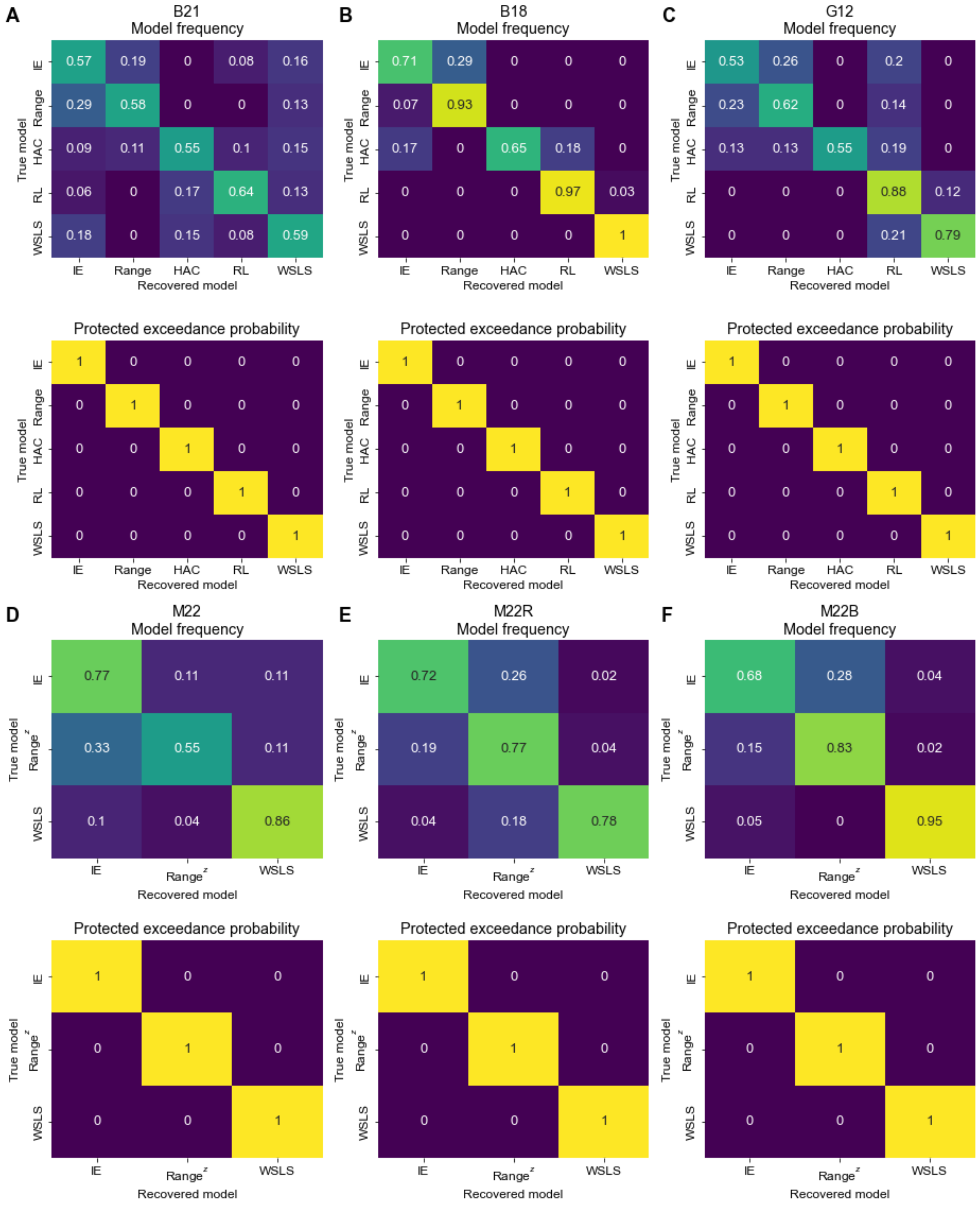


*Fig S7*

## Reviewer 3

**In this study, Molinaro & Collins investigate the computational form of context-dependent reinforcement learning in humans. Over the analysis of previously published datasets (from 2 different research groups), ex-ante simulations on unpublished data, and the analysis of a new dataset using a similar - yet novel - reinforcement learning task, the authors compare different candidate models implementing context-dependent learning in different forms (mainly, intrinsically enhanced and range adaptation models). Model fitting, comparison and falsification slightly favored the intrinsically enhanced model over other candidates in most of the datasets. Overall, this represents interesting work, clear and well written. Strengths of the paper include the re-analysis of several datasets coupled with ex-ante simulations AND a novel task, as well as several candidate normalization models. However, I have several major concerns about model implementation and interpretation that, I believe, limit the study's contribution to the literature, in its current state.**

We thank the reviewer for both their positive words and critical comments on our manuscript. Since Reviewer 3 signed her review, we take this opportunity to thank her for making the data for previous tasks available for us to reanalyze, and give us this opportunity to support our new theory, which we see as complementary to the range adaptation theory she, Dr. Palminteri, and colleagues have developed.

**1. Intrinsically enhanced model. The model is defined as a weighted sum between the absolute experienced outcome (which is not really true - see my next point) and a relative measure (whether the outcome is maximum within its own context). Mathematically, the model is equivalent to the already proposed "hybrid" model in B18 to account for partial adaptation - and the results reported here are therefore similar for this dataset (including the analyses on the weight parameter, see B18 Fig 4). Yet, this similarity is only mentioned in the discussion (line 574) and mostly overlooked over the whole paper. This implementation had some major issues that lead us to move to range adaptation: the weighted sum is a descriptive model by nature and not very plausible on a cognitive level. Previous studies with similar paradigms rather support the hypothesis that contextual information is integrated in a single brain region, as partial (or full) adaptation rather than dual valuation (Padoa-Schioppa, 2009; Palminteri et al. 2015; Rigoli et al. 2016; Burke et al. 2016). This evidence, coupled with the dynamic propensity of adaptation over the task time (see B18, Fig 4a), placed range adaptation (as described in B21) as a good candidate model: participants first start encoding the absolute outcome, and as they progressively learn context values, are able to recognize what constitutes the best choice in each context. Here, a lot of assumptions are made implying that participants already know the structure of the task (the context/maximum values are**

**known from the beginning, not learned over trials), which might not be the case (see B18 Fig 4e-f).**

We thank the reviewer for this insight. The reviewer saw a limitation in the model in B18 as being "descriptive [...] and not very plausible on a cognitive level". We view our intrinsically enhanced model as providing a cognitively valid, mechanistic, and plausible theoretical reason for the shared mathematical formulation in the context of B18. Furthermore, as a reviewer noted before, the overlap between the intrinsically enhanced and B18 hybrid models is restricted to tasks with binary choices only, and in which the same outcomes are associated, though with different probabilities, to options within the same context – unlike tasks for the B22, M22, M22R, and M22B data sets. We now make this point clear in the Discussion (lines 649-652), but also mention the mathematical equivalence with a distinct theoretical interpretation earlier in the paper when discussing Results for B18 (lines 246-251).

While we agree with the reviewer that values might eventually be represented as a unitary construct in the brain, the evaluative process is still likely to require the integration of multiple aspects of an outcome. For example, it is known that costs (e.g., in terms of effort or price) and benefits are weighted against each other in brain regions associated with outcome valuation (Croxson et al., 2009). Moreover, separable neural representations have been found for different attributes of an outcome alongside its overall value (Kahnt et al., 2011). It is also not uncommon for computational models to integrate different values in a manner analogous to the one presented for the intrinsically enhanced model. For instance, hybrid model-based and model-free models weigh Q-values from the two components in order to choose among available options (Gläscher et al., 2010).

Regarding the lack of dynamicity, we agree that the current models could be expanded to incorporate more subtle aspects of subjects' learning and decision-making. However, for the sake of simplicity, we do not address the issue in the present article. We note that the same dynamicity the author mentions being present in B21 is absent in the more recent version of the authors' model of range adaptation described in B22. Nonetheless, we now specifically note the lack of dynamicity in our model in the Discussion (lines 610-620).

With all of this in mind, we think the intrinsically enhanced and range adaptation models are not necessarily mutually exclusive, as we now mention more explicitly in the Discussion (lines 629-632). Our point in this article is mainly to propose an alternative view that, coming from a different perspective, might benefit the field.

> *Lines 246-251:* We note that, consistent with our findings, the winning model in (Bavard et al., 2018) was a hybrid between relative and absolute outcome valuation

which, in mathematical terms, was equivalent to the intrinsically enhanced model. However, as discussed below (see Discussion), the theory behind the hybrid model presented in (Bavard et al., 2018) aligns more closely with the range adaptation mechanism, and the mathematical overlap only exists for data set B18.

*Lines* 610-620: In addition to the lack of dynamicity,, we make the simplifying assumptions that different aspects of outcome valuation are combined linearly and that goal-dependent signals are encoded as binary outcomes.

*Lines* 629-632: Along the same line of research, it may be possible to test for range adaptation as an adjunct mechanism to intrinsically enhanced reinforcement learning, as the two systems are not mutually exclusive.

*Lines* 649-652: Crucially, the two models only overlap in specific instances, i.e. tasks in which only binary choices are presented and the same outcomes are associated, though with different probabilities, to options within the same context. This is not the case for the majority of tasks analyzed here.

**2. Commensurability issue. One other arguable feature of the intrinsically enhanced model is that the outcomes have to be rescaled in order for it to make sense. As stated in the methods (line 714), the outcomes are rescaled between -1 and 1 to allow comparison of fit (temperature) parameters. I however disagree with the next sentence (line 717-718): this could be a theoretical claim in the sense that if the values are not rescaled in a task such as B22 or M22 where the outcomes have a much higher magnitude, then the 'absolute' component of the model would overweight the 'relative' one, the latter being always bounded between 0 and 1. Even by assuming that the participants somehow know the outcome boundaries from the beginning of the task, one of the components would have to be rescaled, which is a drawback not implemented in the range adaptation model. 2B. To make the two models comparable, one possibility could be to fit the intrinsically motivated model without assuming the normalization of the absolute outcomes. It could still work by adjusting the weight parameters, but then the authors will have to explain why (inevitably) the weight parameters change radically from an experiment to another on (as a function of the actual magnitude of the rewards at stake; a problem that does not arise with the range adaptation model).**

We agree with the reviewer that the intrinsic reward vs. extrinsic reward commensurability question is both important and not addressed by our model. However, we argue that this does not obviate the usefulness of our model. Below, we unpack all those points, and

explain why we do not think the approach proposed by the reviewer is consistent with our theoretical proposal.

We agree that commensurability is an interesting problem and one that is not directly addressed by our model. As the reviewer points out, we divide extrinsic rewards by an experiment-specific amount so as not to exceed the [-1, 1] range. This puts rewards on the same scale as intrinsic rewards (or the “goal” of each trial), which are arbitrarily set at {0, 1}. We note that this is mathematically equivalent to putting goals on the same scale as numeric outcomes. For instance, let us consider experiment M22 where absolute rewards were within [0, 100]. Here, the intrinsically enhanced model we developed would be equivalent to one where extrinsic rewards are left to their original value and intrinsic rewards belong to {0, 100} – with the caveat that this version would lead to a rescaling of the $\beta$ parameter. While our original formulation permits a more direct comparison between the parameters of different models (which is why we prefer it), the alternative formulation stresses the fact that the commensurability issue does not necessarily stem from a problem of rescaling extrinsic rewards to intrinsic goals, but the opposite. In other words, the commensurability problem arises because so little is known about how the value of achieving a goal is set. Our model assumes that the value of reaching a goal is roughly equivalent to the highest available reward. We do not claim that this simplification holds explanatory power, but consider it the simplest possible assumption based on the heuristic meaning of attaining a goal. As we mention in the Discussion, a very important future direction for research will be to understand how goals are selected and goal-dependent rewards are set. We now re-emphasize this point and mention how it relates to the commensurability issue (lines 625-629).

The suggestion the reviewer is making by proposing to fit the intrinsically enhanced model without assuming the normalization of absolute outcomes does not faithfully represent our theory. It is beyond the scope of this work to understand how different sources of value are made comparable at the implementational level, largely because research on goal setting and representation in the context of decision-making is so limited. Moreover, we do not wish to exclude range adaptation as a possible, parallel mechanism for context-based valuation, a point we now stress in the Discussion (lines 629-632).

We also now clarify that all outcomes were divided by a single rescaling factor per experiment to be between [-1, 1] if negative outcomes were present, or between [0, 1] if outcomes were in the positive range (lines 785-792).

> *Lines 625-629:* A question for future neuroimaging experiments is how the brain may automatically compute such rescaling and escape commensurability issues. As our understanding of how goals are selected and goal-dependent rewards are adjusted

to circumstances, intrinsically enhanced models of behavior could be improved with increased explanatory power.

*Lines* 629-632: Along the same line of research, it may be possible to test for range adaptation as an adjunct mechanism to intrinsically enhanced reinforcement learning, as the two systems are not mutually exclusive.

*Lines* 785-792: All outcomes were divided by a single rescaling factor per experiment to be between [-1, 1] if negative outcomes were present, or between [0, 1] if outcomes were in the positive range, to allow comparison of fit parameters (in particular β) across models. For example, rewards were divided by 10 (for B21) or 100 (for B22 and M22). Note that this is simply a convenient reparameterization, not a theoretical claim - it is mathematically equivalent to a model where the outcome is not rescaled, the goal intrinsic value is equal to the maximum possible extrinsic reward, and β is divided by the same scaling factor.

**2C. I also think it would be important to present in the main text the model comparison results showing that the RANGE model better fits the majority of participants in the replication experiment (Figure S2).**

We thank the reviewer for this suggestion. However, upon addressing an issue in our previous data preprocessing pipeline (see above: Reviewer 1, comment #2), the IEM model provided a better for the majority of the participants, consistent with our original study and the model validation results. Therefore, we decided to leave the replication in the supplementary materials. However, if the reviewer still believes the replication results should be moved to the main text, we are happy to do so.

**3. Range adaptation model predictions. Given the implementation of the range adaptation model in this paper, the model should be able to reverse participants' ability to select the highest EV stimulus in test trials with ΔEV = 1.75 in the experiments where complete feedback is provided during the test phase (B21), but the model simulations suggest otherwise (see Figure 2A). This does not square with the results reported in B21 and rather suggests a potential error in the fitting or the simulations that may not take the feedback provided the test phase into account.**

We thank the reviewer for noticing this inconsistency with previously published results. Indeed, the features of model validation the reviewer refers to were diagnostic of a bug in our code which prevented updated ranges in the test phase to be taken into account. We apologize for not catching this issue earlier. We have now updated our code and re-ran the analyses. The updated results are presented in Fig 2. As can be observed through the figure

and the updated text (lines 180-184), our previous results hold after correcting for this error.

> *Lines 180-184:* While each of the alternative models also provided the best fit in a fraction of the participants (model responsibilities: intrinsically enhanced = 0.34, range adaptation = 0.19, hybrid actor-critic = 0.17, unbiased RL model = 0.11, win-stay/lose-shift = 0.18), the responsibility attributed to the intrinsically enhanced model was highest (Fig 2B).

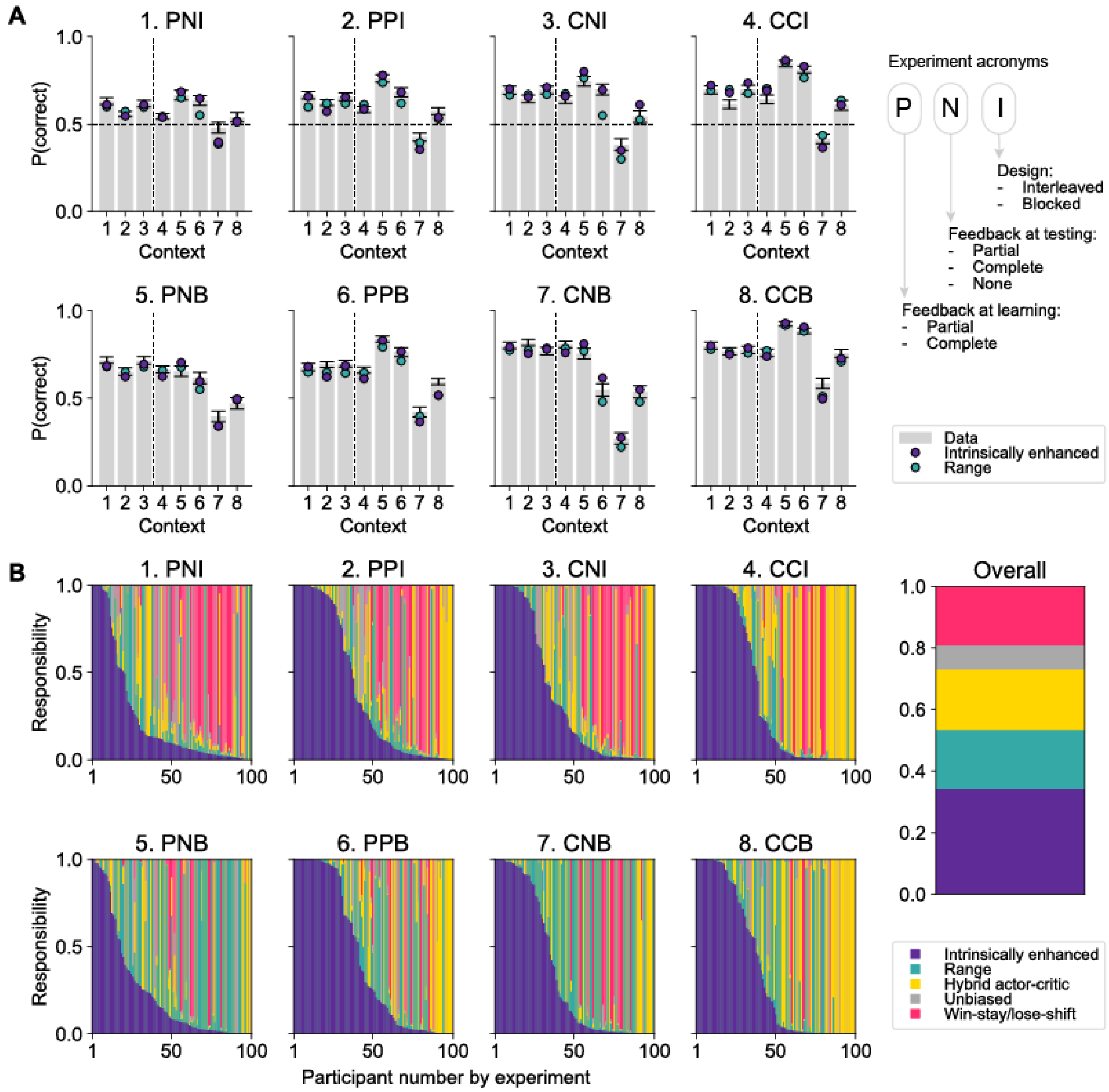


*Fig 2*

**4. Range adaptation implementation. The range adaptation model implemented in the present work differs from that originally presented in B21, in that it assumes that the actual range is perfectly known from the beginning and not learned with a dedicated learning rate (alphaRANGE). The authors mention that this parameter was not included to avoid over penalization and because it was not very well recoverable. However, the fact that the exact value of the parameter is not - or weakly - recoverable does not necessary imply that the additional degree of freedom/flexibility is not recoverable or justified that model space level. In other terms, weak parameter recovery for alphaRANGE could still be associated with good model recovery for a model using the alphaRANGE. This could be the case when, for example, the choice patterns generated by models with alphaRANGE=0.2 and alphaRANGE=0.4 are very similar (which is probably the case: Rmax and Rmin should converge quickly), BUT still quite different from those of a model with alphaRANGE=0.0. Even though I have no reason not to trust the authors concerning the fact that the addition of this parameter penalized the model and made no radically difference, since the goal of the paper is comparative, it would be informative to include this parameter, at least in the Supplementary materials and at least for the where it was originally proposed datasets (B18, B21). This is also important because currently the comparison between the range adaptation model and the intrinsically enhanced model confounds two factors: (1) the architecture (which is what the authors are interesting in) and (2) the fact that the normalization process is assumed to flexible in one case (thanks to the weight parameters) and inflexible in the other case (range adaptation model).**

Upon the reviewer's suggestion, we ran HBI on data from B21 using a version of the range adaptation model that included $\alpha_{range}$ as a mechanism to dynamically update the estimates for minimum and maximum values in each context. In data set B21, the addition of this parameter disadvantaged the range adaptation model compared to the intrinsically enhanced model (Fig S15), such that the responsibility assigned to the former was much weaker than found with simpler implementations (model responsibilities: intrinsically enhanced = 0.50, range adaptation with $\alpha_{range}$ = 0.01, hybrid actor-critic = 0.22, unbiased = 0.07, win-stay/lose-shift = 0.20; Fig S15). The same was true for B18 (model responsibilities: intrinsically enhanced = 0.97, hybrid actor-critic = 0.03). However, since we did not find references to $\alpha_{range}$ in B18, we do not report model comparisons with the $\alpha_{range}$ implementation of the range adaptation model for data set B18 in the manuscript.

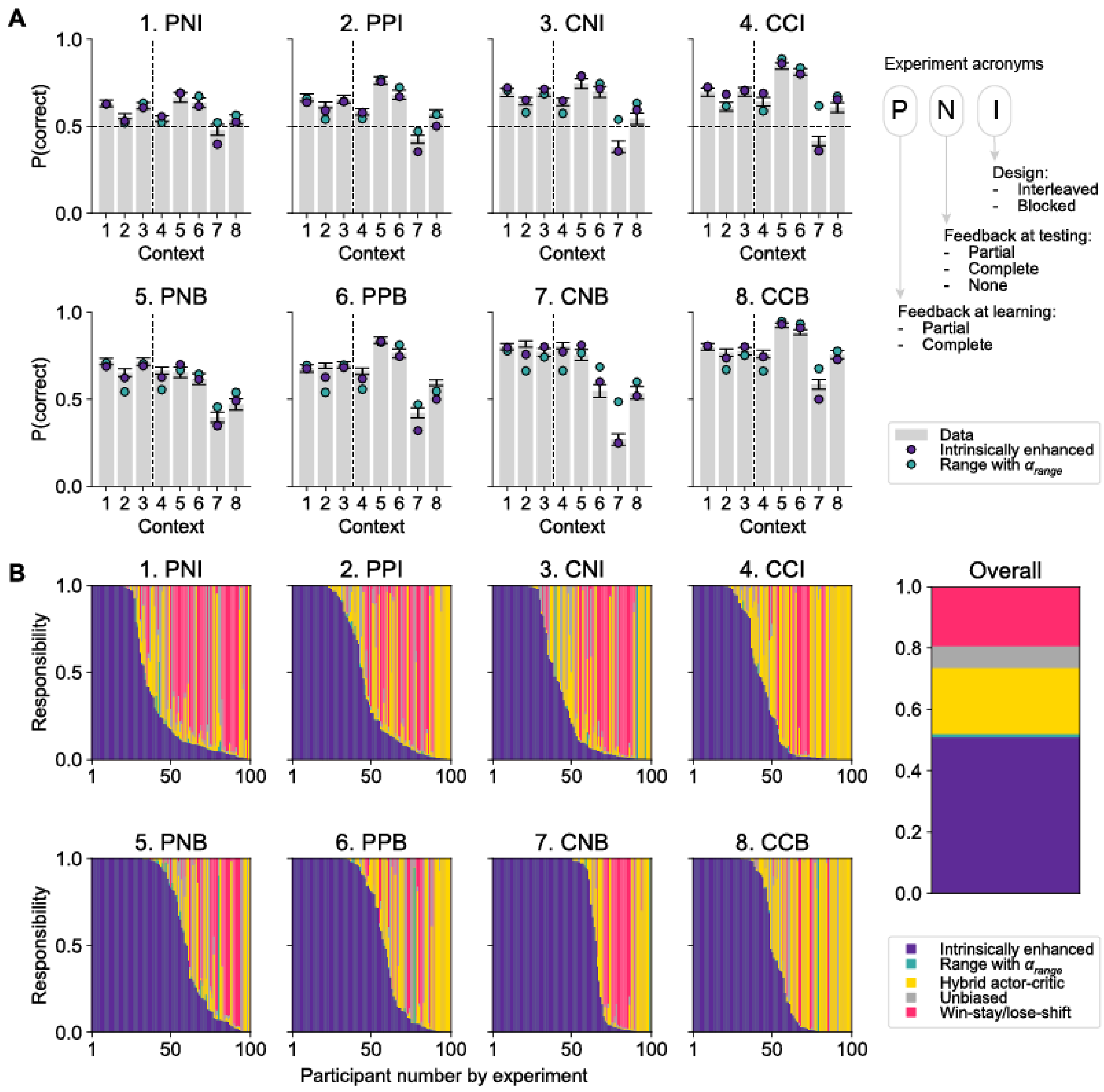


*Fig S15*

**5. Context definition in the novel task. In M22, the reader will benefit from a more precise definition of what constitutes a context. In previous studies, the context would be composed of 2 or 3 alternatives, stable over the learning part of the task. Here, different choice problems (choosing between options A/B/C, or A/B, or A/C) are treated as one context. My understanding is that the contexts values are the ones of the trinary choice, but this might not be the case (see e.g., the debriefing questions in B18 Fig 4e-f, where some participants did not even notice that the options were arranged in fixed pairs). The authors could consider the possibility that if the context is defined by the options it includes, then if the 'trinary maximum' is not there, the context changes and**

**the 'trinary second best' becomes maximum. In such a setting, even a simpler form of range adaptation (such as the one proposed in B22) could make similar predictions as the intrinsically enhanced model.**

We agree with the reviewer that our definition of context was potentially ambiguous and could lead to a confound, a point Reviewer 2 also made. To obviate ambiguity in the definition of context in cases where only two of three options from a choice set were available, we ran an additional study (M22B) controlling this potential confound. Here, instead of hiding unavailable options, we made them visible, but unresponsive to the participants' mouse clicks (M22B; lines 413-425, Text S2, Fig S4). The new results (N=50) confirm our previous behavioral and computational modeling findings (Fig S5-S6).

> *Lines 413-425*: We note that, while previous studies defined contexts as the set of available options at the time of a decision (Bavard et al., 2021; Bavard & Palminteri, 2022) here we adopt a more abstract definition of context, which comprises all the options that tend to be presented together – even when only two of them are available (a feature that was absent from previous task designs). Nonetheless, participants might have interpreted choice sets in which only two out of three outcomes were available as separate contexts. With the latter interpretation, range adaptation and intrinsically enhanced models would make the same predictions. To encourage participants to consider the two sets of stimuli as belonging to one context each (even when only two out of three options from a set were available), we designed a control study (M22B; N=50; Text S2) in which, instead of being completely removed from the screen, unavailable options were simply made unresponsive. Therefore, participants could not select unavailable options, but the stimuli and outcomes associated with them were still visible.

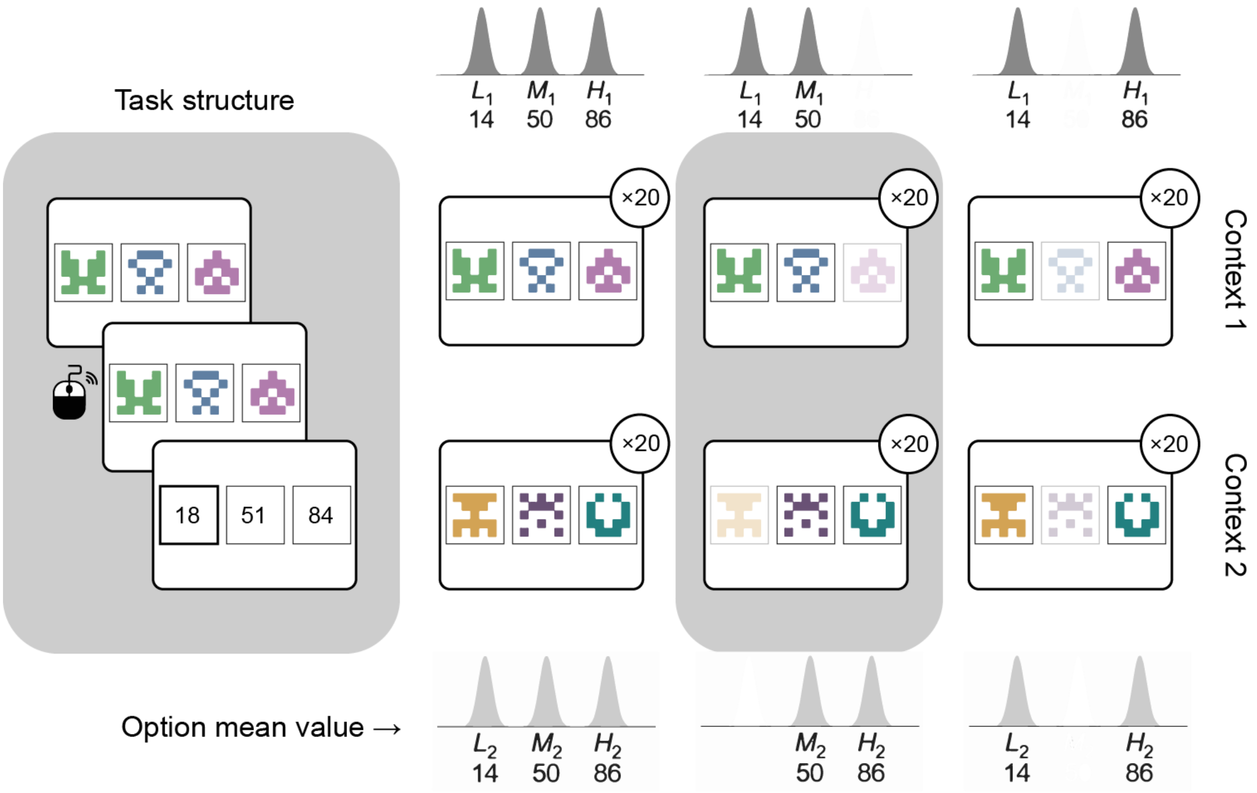
Task structure
18
51
84
Option mean value →
×20
Context 1
Context 2
$L_1$ 14
$M_1$ 50
$H_1$ 86
$L_2$ 14
$M_2$ 50
$H_2$ 86

*Fig S4*

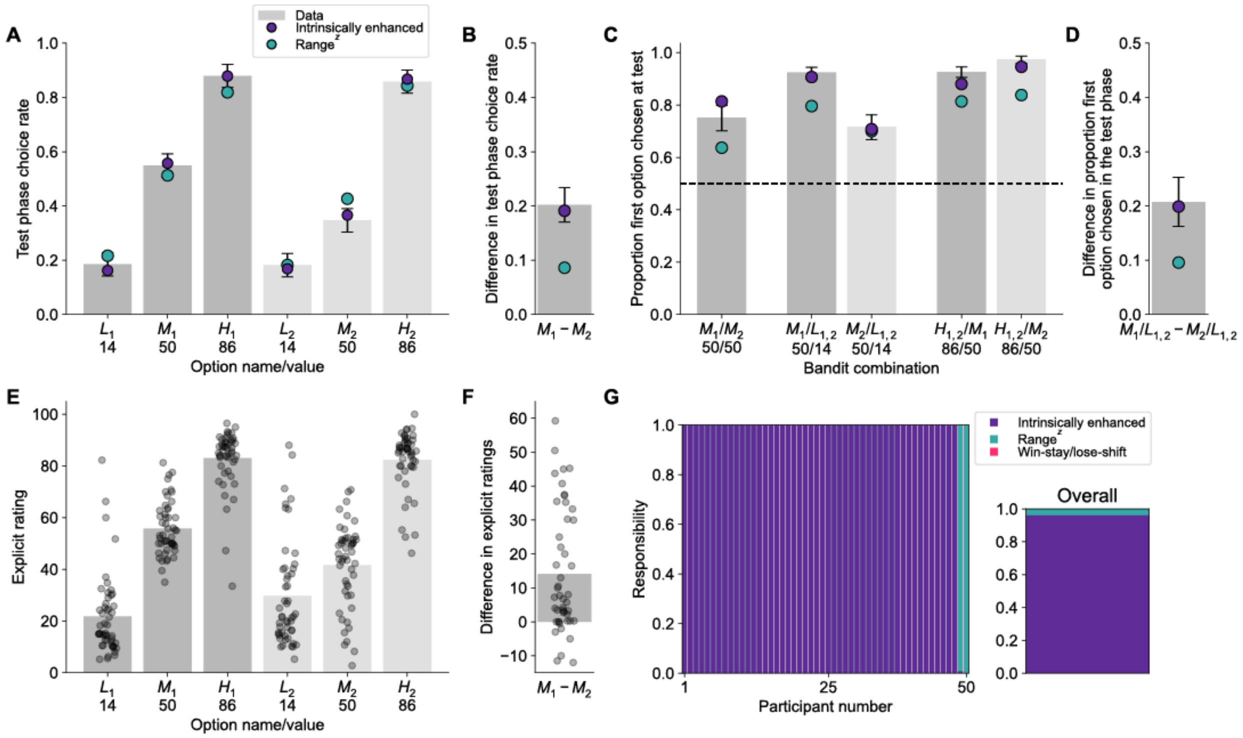
A
Data
Intrinsically enhanced
Range
Test phase choice rate
Option name/value
B
Difference in test phase choice rate
$M_1 - M_2$
C
Proportion first option chosen at test
Bandit combination
$M_1/M_2$ 50/50
$M_1/L_{1,2}$ 50/14
$M_2/L_{1,2}$ 50/14
$H_{1,2}/M_1$ 86/50
$H_{1,2}/M_2$ 86/50
D
Difference in proportion first option chosen in the test phase
$M_1/L_{1,2} - M_2/L_{1,2}$
E
Explicit rating
Option name/value
F
Difference in explicit ratings
$M_1 - M_2$
G
Intrinsically enhanced
Range
Win-stay/lose-shift
Responsibility
Participant number
Overall

*Fig S5*

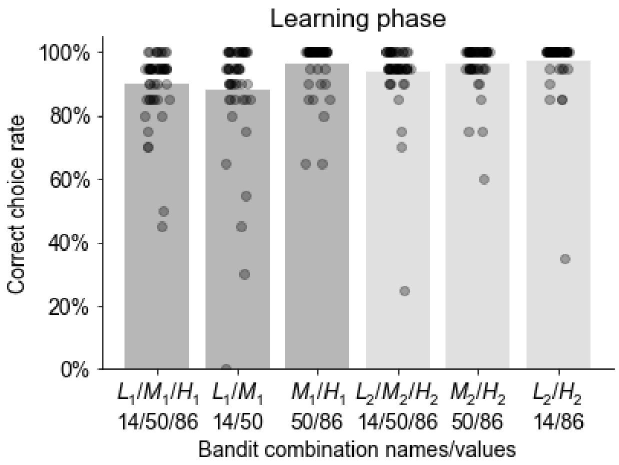


*Fig S6*

**Minor points**

**6. line 712. Is there any reason why the authors chose to fit different temperature parameters for both phases of the task? This suggests that the optimization was performed on the whole dataset, which was not the case of B18 and B21 where it was done on the learning phase data only. Would the intrinsically enhanced model perform equally well in the test phase if this was the case?**

To answer the reviewer's question, we fit different temperature parameters for the learning and the test phase of the task as we believe it is reasonable to expect different noise and exploration levels when the task structure suddenly changes, especially when feedback becomes unavailable. While we agree with the reviewer that fitting on part of the data and validating the model on the rest of it has potential benefits, many of our specific predictions are evident in the test phase. Therefore, our ability to correctly identify the winning model might be reduced if we limit likelihood calculation to the learning phase, where competing models are harder to disentangle. We also note that both the separate temperature parameter for the two phases and the fitting procedure on the whole task are true for the range$^z$ model as well as the intrinsically enhanced model, which should make the comparison between the two models fair.

Nonetheless, we decided to address the reviewer's concern by selecting one of the data sets that were specifically designed to compare the intrinsically enhanced and range models (M22) and calculate each model's responsibility based on its likelihood in the learning phase only. This procedure did not significantly alter the results, as the intrinsically enhanced model still had the highest responsibility across participants (responsibilities were: intrinsically enhanced model = 0.60, range$^z$ = 0.31,

win-stay/lose-shift model = 0.09; plot A below). Moreover, to demonstrate the necessity of separate temperature parameters for the learning and the test phase by comparing 1) the complete intrinsically enhanced model, as presented in the main text, 2) a version of the intrinsically enhanced model that shares the temperature parameter across task phases, and 3) the win-stay/lose-shift model (to capture variability in participants' behavior; Piray et al., 2019). By running this analysis, we confirm that the full model outperforms the simpler one, supporting our case for separate βs for the learning and test phases (responsibilities were: intrinsically enhanced model = 0.65, intrinsically enhanced model with 1 β = 0.35; plot B below). We now make a brief note of these results in the Methods (lines 917-919).

> *Lines 917-919:* Data were fit to both the learning and test phases. In M22, we confirmed that fitting to only the learning phase did not change results and that phase-specific temperature parameters (βs) improved fit.

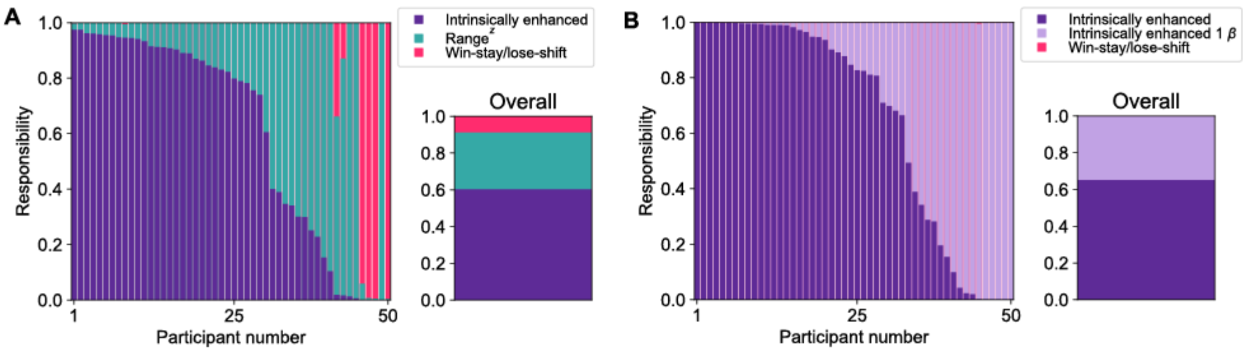


*Model comparison results obtained by fitting only on the learning phase (A) and by comparing the full intrinsically enhanced model to one with only one β.*

**7. line 733. Separating learning rates between conditions also makes the assumption that the participants have a clear understanding of the task structure. Alternative implementations include separating learning rates with the sign of the prediction error, which should not produce a huge difference in the results.**

Our decision to have separate learning rates for contexts of gain vs. losses was based on the previous literature showing that positive and negative outcomes are processed differently in the brain (Palminteri & Pessiglione, 2017). While it is true that this separation of learning rates assumes participants have grasped which stimuli correspond to gain versus losses contexts, we would expect such an understanding to occur early on in the experiment. Moreover, the model does not inherently imply that the two learning rates should be different.

Nonetheless, following the reviewer's suggestion, we implemented a version of our models with two separate learning rates based on the sign of the prediction error. We did this for both B18 and G12, where we use two separate learning rates for positive and negative outcomes. This negatively impacted range adaptation compared to our initial results, as shown in the model validation and comparison plots below (B18 model responsibilities: intrinsically enhanced = 0.97, hybrid actor-critic = 0.03; G12 model responsibilities: intrinsically enhanced = 0.47, range$^z$ = 0.36, hybrid actor-critic = 0.07, unbiased = 0.10). Presumably, such change could be explained by the fact that range adaptation mechanisms make negative prediction errors rare, meaning that the negative reward prediction error learning rate was rarely used. Therefore, the use of separate learning rates described by the reviewer led to unwarranted complexity, at least for range adaptation. For these reasons, we decided to leave the model as specified in our initial submission.

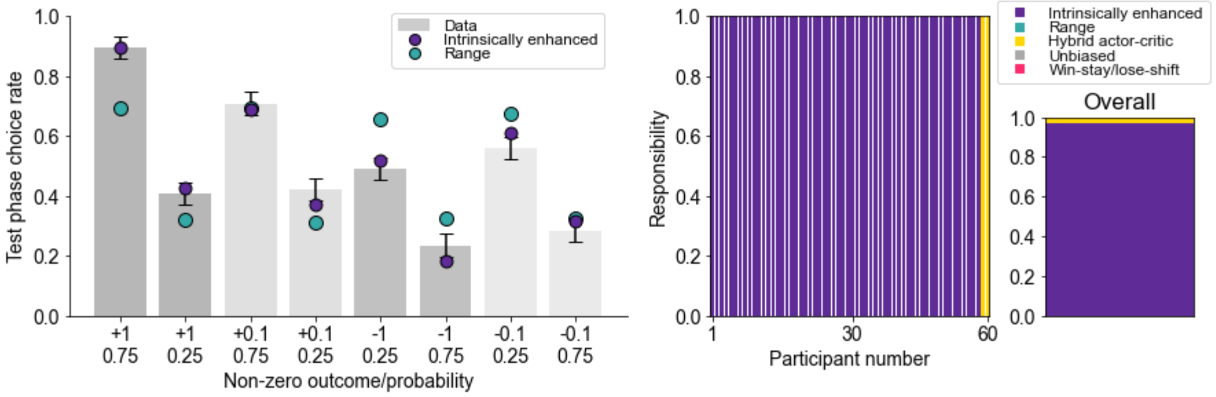


*Model validation and comparison results using separate learning rates for positive and negative RPEs in B18*

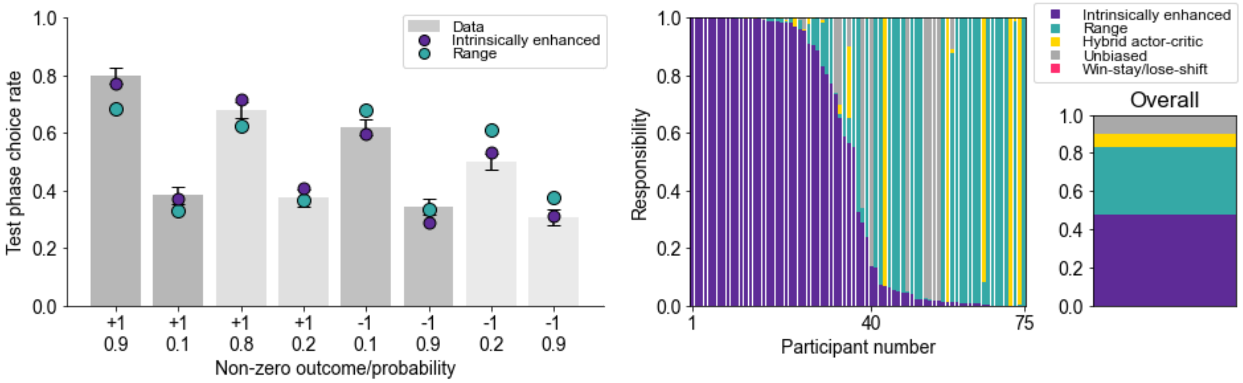


*Model validation and comparison results using separate learning rates for positive and negative RPEs in G12*

**8. line 751. The division by null values was avoided in previous studies by adding 1 to the denominator, which also allowed the models to be nested within each other.**

The author is correct. However, the addition of a +1 at the denominator caused the model to never fully normalize. If we consider an example in which the current context's range is 0-1, and the participant gets an outcome of 1, a range model constructed as the reviewer describes would make the subjective reward equal to

$$\frac{1-0}{1-0+1} = \frac{1}{2} = 0.5$$

rather than 1, as the theory of the model suggests.

**9. Hayes and Wedell (2022) recently showed that context effects can be "top-down" modulated by focusing the participants attentions on internal or external features; Juechems et al. (2022) recently showed that context effect can be modulated "bottom up" by implicitly instructing subjects of the benefit of absolute encoding. It would be nice if the authors discuss these results in the light of the intrinsically motivated model.**

We agree with the reviewer that both Hayes and Wedell's (2022) and Juechems and colleagues' (2022) findings are relevant to the Discussion of the intrinsically motivated model. In fact, we had mentioned both studies in the initial submission (now at lines 537-551) and suggested that adaptive responses to task-contingent expectations can be easily captured by an adjustment of the ω parameter depending on participants' expectations or focus.

> *Lines 537-551:* Attentional biases and task demands could also affect the relative contribution of extrinsic and intrinsic reward signals in outcome valuation. For instance, Hayes & Wedell (2022) manipulated attentional focus in a reinforcement learning task by asking participants to rate, at occasional time points in the task, either how they felt about particular options or the reward amount they expected to be associated with particular options. The latter condition resulted in blunted context-sensitive valuation during the transfer portion of the task, which may be formalized as a greater contribution of absolute reward values (i.e., the term participants' attention was brought towards) compared to internally rewarding features of the evaluated options (which, by contrast, was emphasized in the former condition). Similarly, Juechems et al. (2022) found that, following a learning phase in which participants made within-context choices, exposure to decision sets that combined previously experienced contexts resulted in subsequent reductions in context-sensitive valuation. This behavior is thought to result from an adaptive response to task-contingent expectations and can be easily captured by an adjustment of the ω parameter once participants expect to make cross-context decisions.
