# Supplementary material for: Intrinsic rewards explain context-sensitive valuation in reinforcement learning
Source: PLoS Biol. 2023 Jul 17;21(7):e3002201. doi: 10.1371/journal.pbio.3002201 (PMC10374061; doi:10.1371/journal.pbio.3002201)
Supplement: Attachment — Submitted filename: Context RL Revision 2 Response PLOS Bio.pdf [file pbio.3002201.s021.pdf]

We would like to thank the editorial team for their thorough assessment of our manuscript. We have taken all requests into account, and hope the article is now ready for publication at PLOS *Biology*. Below, we address all changes made to the manuscript in response to the editorial requests, which we report verbatim (in bold) for ease of reference.

EDITORIAL REQUESTS:

1) FINANCIAL DISCLOSURES: I noticed in your financial disclosure that you report having received no specific funding for this work. Even if you did not have a grant specifically written to fund this project, we would require that you acknowledge the sources of money that funded this work, including salaries, etc. You should also acknowledge institutional funding or private grants as well, if relevant.

- Following the editor's guidelines, we have updated our financial disclosure statement in the editorial manager. We now specify that we were supported by a Regents Fellowship for Graduate Study from the University of California, Berkeley (GM), and grants from the National Institutes of Health (NIH-R01MH118279) and the National Science Foundation (NSF 2020844; both awarded to AGEC).

2) ETHICS STATEMENT: Please update your ethics statement, in the methods section, to include an approval number. Please also indicate whether the experiments were conducted according to the principles expressed in the Declaration of Helsinki.

- As requested by the editor, we have updated our ethics statement, which now includes an approval number (2016-01-8280). We also indicate that experiments were conducted according to the principles expressed in the Declaration of Helsinki.

3) DATA AVAILABILITY: Thank you for depositing your data on OSF - for some reason I could not access this data to check that it meets our requirements for data sharing. Can you please provide me a reviewer token, or accept my request to view the data? **Please also ensure that figure legends in your manuscript include information on where the underlying data can be found (by referencing the relevant OSF links).

For more information on our data availability requirements, see here: http://journals.plos.org/plosbiology/s/data-availability. Also see this editorial: http://dx.doi.org/10.1371/journal.pbio.1001797

- Access to the OSF repository was granted upon receiving the editorial team's email (4/28/2023). Since then, all OSF repositories have been made available. As a result, we have also updated the respective web addresses by removing the view-only parameter in the manuscript.
- Figure legends in the manuscript now reference the relevant repository links.

As you address these items, please take this last chance to review your reference list to ensure that it is complete and correct. If you have cited papers that have been retracted, please include the rationale for doing so in the manuscript text, or remove these references and replace them with relevant current references. Any changes to the reference list should be mentioned in the cover letter that accompanies your revised manuscript.

- We thank the editor for this opportunity to revise our reference list. No additions, substitutions, or deletions were made. Minor changes in the reference list – all reflected in the track-changes file attached to this submission – were limited to 1) typographical changes to ensure consistent casing in journal titles, 2) formatting changes to ensure compliance with *PLOS Biology*'s citation requirements (i.e., Vancouver citation style), and 3) insertion of citation information (e.g., publisher, issue number) where previously missing.

Lastly, we would like to note that we made minor modifications to some of the figures (e.g., inserting axis labels where missing, aligning tick labels to the correct location, etc.) that do not affect the style or content of previously submitted figures.

Once again, we would like to express our gratitude to the editorial team for their continued interest and support. We remain available for further updates and clarifications, which we hope will help facilitate the publication process at *PLOS Biology*.
