# Supplementary material for: Intrinsic rewards explain context-sensitive valuation in reinforcement learning
Source: PLoS Biol. 2023 Jul 17;21(7):e3002201. doi: 10.1371/journal.pbio.3002201 (PMC10374061; doi:10.1371/journal.pbio.3002201)
Supplement: Attachment — Submitted filename: Context RL Revision 3 Response PLOSBio.pdf [file pbio.3002201.s022.pdf]

We would like to sincerely thank Reviewer 3 (Dr. Sophie Bavard) for her careful investigation of our results. We indeed identified and corrected a code error. We note that this bug only affected new results requested in the revision, in a single figure (Fig. S15), and did not affect the conclusion of this new analysis. Before expanding below, we report Dr. Bavard's comments in full (bold) for ease of reference, as they were sent to us by the Editor.

Thank you for letting me know and for sharing the rebuttal letter. I have taken the time to read it and I appreciate the authors' response to my concerns. However, I would have liked to have an opportunity to engage further with the authors regarding some analytical choices that I think could still be improved

I would like to bring to the academic editors' attention a potential issue with the simulations presented in the rebuttal letter. Specifically, the authors' response to my 4th point is much likely to be invalid, as the simulations seem to suffer from the same code bug found in the previous ones (which was correctly identified and highlighted in my 3rd point). This claim can be easily checked by noting how the presented simulations of the RANGEalpha model (which is supposed to be exactly the same as our model published in Science Advances in 2021) differ from our own published simulations, despite declaring very similar methods (Figure S15 in the rebuttal letter). I believe such a potential mistake should be carefully examined to ensure the accuracy of the comparison between the two models.

Prompted by these comments, we investigated our model fitting and simulation codes and, indeed, have identified the suggested bug in the range model with the alpha_range parameter, which we only used in supplementary analyses at the request of Reviewer 3, as we had previously established internally that the alpha_range parameter over-penalized the range model. The bug has now been corrected, and resulting simulations with the range with alpha_range model match Bavard et al. (2021) much more closely, as we hope Dr. Bavard will agree. For ease of comparison, we present our corrected Fig. S15 next to Bavard et al.'s (2021) Fig. 4D (which we invite you to compare to context 8 across experiments in our panel A). We note that the match is not perfect, as expected. As we have flagged before, we use slightly different model simulation and model fitting techniques, as we have explained and justified in our previous response. Thus, slight deviations are expected; nonetheless, the new simulations match qualitatively, and capture the behavior adequately, similarly to Bavard et al. (2021), confirming that the bug we overlooked has been fixed.

Bavard et al., 2021 – Fig. 4D

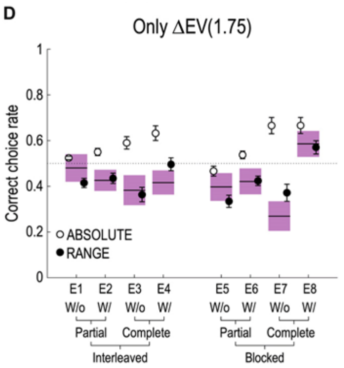

Molinaro & Collins – Fig. S15

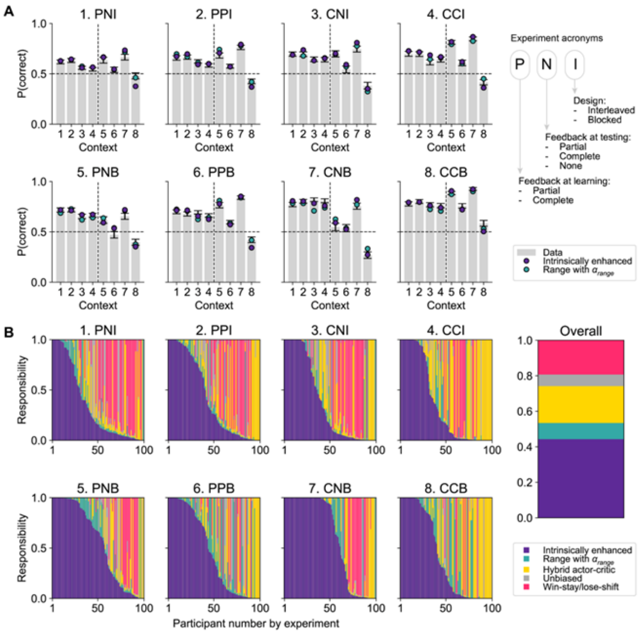

Left: Results from context 8 (with ΔEV = 1.75) of data set B21 from Figure 4, panel D of Bavard et al. (2021). Participants' data (pink boxes indicate the mean and 95% confidence interval) is overlaid with simulations from the range adaptation model (in black). **Right**: our corrected Fig S15. A) Participants' behavior (grey bars) is overlaid with the range adaptation model with alpha_range (in teal), which closely matches the simulations in the original paper in context 8 across experiments. B) Adding alpha_range to the range model does not improve its responsibility.

With the bug fixed, our conclusions are unchanged. Specifically, we still observe that introducing alpha_range penalizes the range model (compared to its simpler instantiation used throughout the article). In addition, we note that Bavard and colleagues also did not include the alpha_range parameter in a more recent implementation of their very own range adaptation model (Bavard et al., 2022). That said, we appreciate Dr. Bavard's insight and the opportunity to correct Fig. S15 before the manuscript is published.

Once again, we are thankful to Dr. Bavard and the editorial team for carefully examining our manuscript and giving us the opportunity to improve its quality, and we apologize for the oversight of this bug in supplementary figure Fig. S15.
